# New SHIVs and Improved Design Strategy for Modeling HIV-1 Transmission, Immunopathogenesis, Prevention, and Cure

**DOI:** 10.1128/JVI.00071-21

**Published:** 2021-05-10

**Authors:** Hui Li, Shuyi Wang, Fang-Hua Lee, Ryan S. Roark, Alex I. Murphy, Jessica Smith, Chengyan Zhao, Juliette Rando, Neha Chohan, Yu Ding, Eunlim Kim, Emily Lindemuth, Katharine J. Bar, Ivona Pandrea, Cristian Apetrei, Brandon F. Keele, Jeffrey D. Lifson, Mark G. Lewis, Thomas N. Denny, Barton F. Haynes, Beatrice H. Hahn, George M. Shaw

**Affiliations:** a Department of Medicine, Perelman School of Medicine, University of Pennsylvania, Philadelphia, Pennsylvania, USA; b Department of Pathology, School of Medicine, University of Pittsburgh, Pittsburgh, Pennsylvania, USA; c Division of Infectious Diseases, Department of Medicine, School of Medicine, University of Pittsburgh, Pittsburgh, Pennsylvania, USA; d AIDS and Cancer Virus Program, Frederick National Laboratory for Cancer Research, Frederick, Maryland, USA; e Bioqual, Inc., Rockville, Maryland, USA; f Duke Human Vaccine Institute, Duke University School of Medicine, Durham, North Carolina, USA; g Department of Medicine, Duke University School of Medicine, Durham, North Carolina, USA; h Department of Microbiology, Perelman School of Medicine, University of Pennsylvania, Philadelphia, Pennsylvania, USA; Emory University

**Keywords:** AIDS, SHIV, human immunodeficiency virus, simian immunodeficiency virus

## Abstract

Previously, we showed that substitution of HIV-1 envelope (Env) residue 375-Ser by bulky aromatic residues enhances binding to rhesus CD4 and enables primary HIV-1 Envs to support efficient replication as simian-human immunodeficiency virus (SHIV) chimeras in rhesus macaques (RMs). Here, we test this design strategy more broadly by constructing SHIVs containing 10 primary Envs corresponding to HIV-1 subtypes A, B, C, AE, and AG. All 10 SHIVs bearing wild-type Env375 residues replicated efficiently in human CD4^+^ T cells, but only one replicated efficiently in primary rhesus cells. This was a subtype AE SHIV that naturally contained His at Env375. Replacement of wild-type Env375 residues by Trp, Tyr, Phe, or His in the other nine SHIVs led to efficient replication in rhesus CD4^+^ T cells *in vitro* and *in vivo*. Nine SHIVs containing optimized Env375 alleles were grown large-scale in primary rhesus CD4^+^ T cells to serve as challenge stocks in preclinical prevention trials. These virus stocks were genetically homogeneous, native-like in Env antigenicity and tier 2 neutralization sensitivity, and transmissible by rectal, vaginal, penile, oral, or intravenous routes. To facilitate future SHIV constructions, we engineered a simplified second-generation design scheme and validated it in RMs. Overall, our findings demonstrate that SHIVs bearing primary Envs with bulky aromatic substitutions at Env375 consistently replicate in RMs, recapitulating many features of HIV-1 infection in humans. Such SHIVs are efficiently transmitted by mucosal routes common to HIV-1 infection and can be used to test vaccine efficacy in preclinical monkey trials.

**IMPORTANCE** SHIV infection of Indian rhesus macaques is an important animal model for studying HIV-1 transmission, prevention, immunopathogenesis, and cure. Such research is timely, given recent progress with active and passive immunization and novel approaches to HIV-1 cure. Given the multifaceted roles of HIV-1 Env in cell tropism and virus entry, and as a target for neutralizing and nonneutralizing antibodies, Envs selected for SHIV construction are of paramount importance. Until recently, it has been impossible to strategically design SHIVs bearing clinically relevant Envs that replicate consistently in monkeys. This changed with the discovery that bulky aromatic substitutions at residue Env375 confer enhanced affinity to rhesus CD4. Here, we show that 10 new SHIVs bearing primary HIV-1 Envs with residue 375 substitutions replicated efficiently in RMs and could be transmitted efficiently across rectal, vaginal, penile, and oral mucosa. These findings suggest an expanded role for SHIVs as a model of HIV-1 infection.

## Abstract (precis)

SHIV infection of Indian rhesus macaques is an important animal model for studying HIV-1 transmission, prevention, immunopathogenesis, and cure. Such research is timely, given recent progress with active and passive immunization and novel approaches to HIV-1 cure.

## INTRODUCTION

Simian-human immunodeficiency virus (SHIV) infection of Indian rhesus macaques (RMs) is an important outbred animal model for studying HIV-1 transmission, prevention, immunopathogenesis, and cure (1–3). Such research is especially timely, given recent progress with active and passive immunization (4–11) and novel approaches to HIV-1 cure (https://www.niaid.nih.gov/diseases-conditions/hiv-cure-research) (12–18), all of which can benefit from rigorous testing and iterative refinement in animal models. Given the multifaceted roles of HIV-1 envelope (Env) in cell tropism and virus entry, and as a target for neutralizing and nonneutralizing antibodies, the particular features of HIV-1 Envs that are selected for SHIV construction and analysis are of paramount importance. This is especially true for vaccine studies designed to administer (10, 11) or elicit (6, 19, 20) broadly neutralizing antibodies (bNAbs).

SHIVs have a long history dating to 1992, when Sodroski and colleagues first subcloned the *tat*, *rev*, and *env* sequences of HIV-1 HXB2c into SIVmac239 (21). This clone was further modified by substitution of the *env* from the dual CCR5/CXCR4 tropic HIV-1 89.6 strain and later adapted by serial passage in RMs, eventually yielding the molecular clone SHIV-KB9 (22). Thus, the earliest SHIVs contained T-cell-line-adapted, *in vivo*-passaged HIV-1 Envs that were CXCR4 tropic, highly syncytium inducing, and cytopathic, and led to accelerated disease in monkeys. As a consequence, many of the essential features of HIV-1 biology, including cell and tissue tropism, sensitivity to neutralizing antibodies (NAbs), immunopathogenesis, transmission efficiency, and natural history were not faithfully represented in the macaque model (3). Attempts to develop a SHIV infection model that included primary (non-T-cell-line adapted) CCR5-tropic Envs were generally met with failure, and when they were successful, such SHIVs often required adaptation by serial monkey passage to achieve consistent replication *in vivo* (3, 23–25). In an attempt to better understand restrictions to SHIV infection and replication in RMs, Overbaugh and Sawyer examined the affinity of primary HIV-1 Envs to rhesus CD4 (26, 27). They discovered that the Envs of most primary HIV-1 strains exhibited low affinity for rhesus CD4 and did not support efficient virus entry into rhesus cells. Overbaugh identified a key amino acid at position 39 in domain 1 of rhesus CD4 that differed between human and rhesus CD4 and was largely responsible for the poor binding and infectivity of primary HIV-1 Envs in rhesus cells (27). This presented a major obstacle to new SHIV designs. Hatziioannou identified a mutation at residue 281 in the CD4-binding region of HIV-1 Env that occurred commonly in SHIV-infected RMs, where it could be shown to facilitate virus replication (28). However, unlike the Env375 substitution, the 281 substitution on its own was unable to consistently convert primary or transmitted/founder (T/F) Envs, which fail to replicate efficiently in RMs, to do so. Moreover, the addition of the 281 mutation to SHIV Envs that already contain a rhesus-preferred Env375 allele did nothing to further enhance virus replication in rhesus animals (29).

We noted from studies by Finzi and Sodroski (30) that residue 375 in the CD4-binding pocket of primate lentiviral Envs was under strong positive evolutionary pressure across the broad spectrum of primate lentiviruses. These investigators further showed that substitution of 375-Ser (found in most HIV-1 group M viruses) by 375-Trp (found in most SIV strains from lower primates) favored an HIV-1 Env conformation that was closer to the CD4-bound state (31–34). Based on these findings, we hypothesized that residue 375 might act as a “molecular switch” conferring enhanced Env affinity to rhesus CD4 (35) and a lower energetic barrier to conformational change following CD4 binding (31, 34, 36, 37) when the naturally occurring Ser or Thr residues were substituted by bulky aromatic residues like Trp. In testing this hypothesis, we discovered that substitution of a single residue, 375-Ser, in primary or T/F HIV-1 Envs by Trp, Phe, Tyr, His, or Met resulted in SHIVs that exhibited enhanced binding to rhesus CD4, increased infection of primary rhesus CD4^+^ T cells in culture, and consistent infection and replication by SHIVs in RMs *in vivo* (35). Importantly, these amino acid substitutions at residue 375 did not alter the tier 2 neutralization phenotype of the primary Envs, nor did they appreciably alter their sensitivity to bNAbs that targeted any of the canonical bNAb recognition sites, including CD4bs, V2 apex, V3 high mannose patch, or membrane proximal external region (35). Thus, it became possible, for the first time, to prospectively design SHIVs that expressed particular primary or T/F Envs, including those that elicited bNAbs in HIV-1-infected humans, and to explore parallels in the immune responses of rhesus monkeys and humans to essentially identical Env immunogens (38). This EnvΔ375 design strategy also made possible the development of SHIVs to evaluate preclinical efficacy of novel active or passive vaccination regimens against challenge by viruses bearing homologous or heterologous primary Envs (7–10). Here, we extend this work by constructing 10 new SHIVs, each containing a strategically selected primary HIV-1 Env, that we then validate for retention of native antigenicity, tier 2 neutralization sensitivity, and efficient replication in human and rhesus CD4^+^ T cells *in vitro* and in RMs *in vivo*. We next describe the development and characterization of a panel of nine SHIV challenge stocks, each containing a unique tier 2 primary HIV-1 Env and grown large scale in primary rhesus CD4^+^ T cells, for distribution as challenge strains for active or passive vaccine protection trials. We show that these SHIVs can be efficiently transmitted by different mucosal routes (rectal, vaginal, penile, or oral) and that current vaccination regimens and passively administered bNAbs can prevent transmission of these viruses at neutralization titers similar to those reported in the recently concluded human antibody-mediated prevention (AMP) trials (11). Finally, we describe a new second-generation design strategy that simplifies SHIV construction and eliminates extraneous *tat1* and *env* sequences, thereby making the rhesus-SHIV infection model a more readily accessible and useful research tool.

## RESULTS

Ten primary HIV-1 Envs were chosen for SHIV constructions (Table 1A). These Envs were selected based on their genetic subtypes, biophysical properties, derivation from primary or T/F virus strains, and, in some cases, prior development as candidate vaccine strains for human clinical trials (see Table 1 for Env features and relevant literature citations). Env subtypes included A, B, C, AE, and AG, which complement subtype A, B, C, and D SHIVs that we reported previously (see references 35 and 38 and Table 1B). All 10 of the new SHIVs contained Envs from tier 2 viruses except for Q23.17 Env (39), which has been variably classified as tier 1b or 2 (40–42). Seven of the new SHIVs contained Envs from T/F strains of HIV-1. The 1086 Env (43) corresponds to a vaccine strain employed in the HVTN 703 efficacy vaccine trial (44–46), and the B41 Env was developed as a SOSIP trimer for potential human immunizations (47). The CE1176 Env is from a widely used global test panel for bNAb detection (41). Env RV217.40100 is a new subtype AE T/F strain (48, 49) and Envs CH1012 and CH0694 are T/F strains that elicited potent bNAbs in their respective human hosts (50, 51). Envs T250-4, ZM233, WITO, Q23.17, and CAP256SU were shown previously to bind unmutated common ancestors (UCAs) of human V2 apex targeted bNAbs (52–54). Thus, the Envs selected for new SHIV constructions exhibited unique pedigrees complementary to previous SHIV designs (35, 38, 55–64) that made them desirable for downstream investigations related to HIV-1 transmission, prevention, immunopathogenesis, or cure.

**TABLE 1:** Genetic and biological features of HIV-1 Envs used for new and previous SHIV constructions

HIV-1 env	Subtype	Env GenBank accession no.	SHIV GenBank accession no.	Env properties	References
New SHIV constructions					
Q23.17	A	AF004885	MW410736	Cloned from primary isolate; binds V1V2 bNAb UCAs	39, 52–54
WITO4160	B	FJ496176	MW410737	T/F^*a*^; binds V1V2 bNAb UCAs	52–54, 73
B41^*b*^	B	EU576114	MW410732	T/F; SOSIP immunogen	47, 73
CE1176	C	FJ444437	MW410733	T/F; global neutralization panel	41, 43
CH1012	C	MG898887	MW410734	T/F; elicited bNAbs in human	50
ZM233	C	DQ388517	MW410738	Cloned from primary isolate; binds V1V2 bNAb UCAs	52–54, 64
1086	C	FJ444395	MW410739	T/F; P5 vaccine trial	43–46
CH0694	C	KJ700458	MW410741	T/F; elicited bNAbs in human	50, 51
RV217.40100	AE	MN792078	MW410740	T/F; Thai AE subtype	48, 49
T250-4^*c*^	AG	MW507842	MW410735	Primary isolate; binds V1V2 bNAb UCAs	20, 52–54
Previous SHIV constructions					
BG505^*d*^	A	DQ208458	KU958484	T/F; elicited bNAbs in human; SOSIP immunogen	55, 56, 61
YU2	B	M93258	KU958489	Macrophage-tropic; brain-derived	57, 58
CH505	C	KC247556	KU958487	T/F; elicited bNAbs in human	59
CH848	C	KX216883	KU958488	T/F; elicited bNAbs in human	60
CAP256SU	C	KF241776	MT509359	T/F; binds V1V2 bNAb UCAs	52–54, 62
191859	D	JX203061	KU958486	T/F; macrophage-tropic	63

a T/F denotes transmitted/founder viral genomes, as reported in the references cited.

b B41 Env is also designated 9032.08_A1 (73).

c T250-4 is one of several *env* molecular clones from the isolate CRF_AG_250. One of these clones was designated by Ellenberger and colleagues as “250” and contributed to the NIH HIV Reagent Program (catalog number 11594) and to GenBank (accession number EU513189). This “250” *env* clone number EU513189, when expressed in 293T cells and used to pseudotype an *env*-minus HIV-1 proviral backbone, yields noninfectious virions in the TZM-bl assay (G.M.S., unpublished). The T250-4 *env* clone (GenBank accession number MW507842) differs from number EU513189 by six nucleotides and three amino acids and yields highly infectious HIV-1 Env-pseudotyped virions and highly infectious SHIV.T250-4 virions (see Table 2).

d SHIV.BG505 exists in two versions with and without an asparagine and potential *N*-linked glycan at Env residue 332 (35).

**FIG 1:**
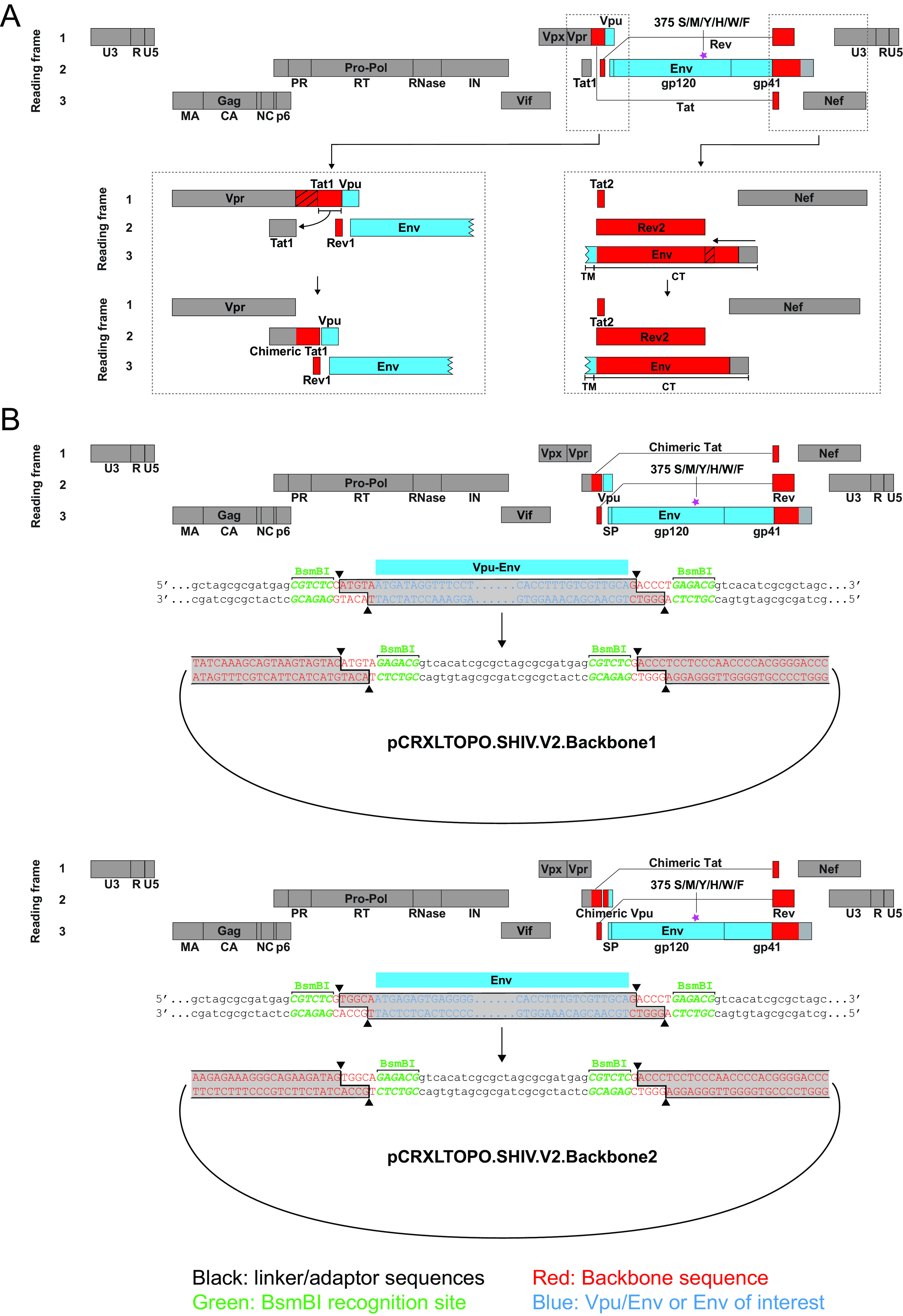
First (A) and second (B) generation design schemes for SHIV constructions. The first generation design (35) consists of a proviral backbone of SIVmac766 (a T/F clone derived from the SIVmac251 isolate), shown in gray, and HIV-1.D.191859, shown in red. A *vpu-env* amplicon is subcloned into this plasmid vector as described (35). Note in the expanded figures in panel A that the proviral backbone design results in duplications of SIV and HIV-1 *tat1* and gp41 sequences (indicated by slash marks) that are spontaneously deleted during *in vivo* replication of the SHIVs (Fig. 2). The second-generation design scheme (panel B) eliminates these duplications and adds a BsmBI cloning site that allows for simple introduction of either a *vpu-env* amplicon or an *env* amplicon.

**FIG 2:**
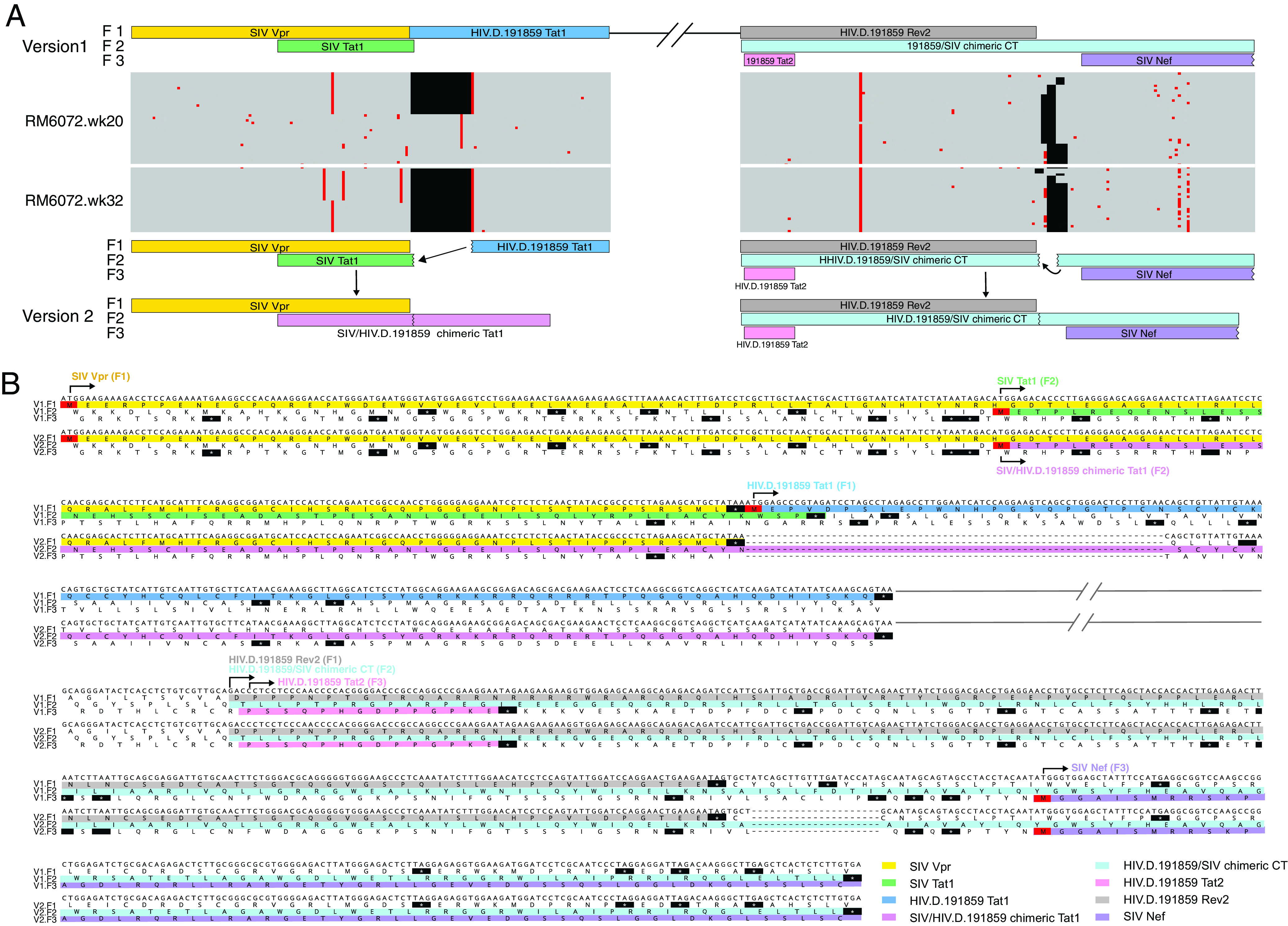
Spontaneous deletion of redundant *tat1* and *env* gp41 sequences from the version 1 SHIV proviral backbone in SHIV.C.CH505-infected monkey RM6072. (A) Expanded segments of the *vpr- tat1* gene overlap and the *tat2-rev2-env-nef* gene overlap from Fig. 1A are illustrated above Pixel plots (https://www.hiv.lanl.gov/content/sequence/pixel/pixel.html) of 39 single genome sequences from RM6072 at 20 weeks post-SHIV infection and 26 sequences at 32 weeks post-SHIV infection. A 68-bp deletion of redundant sequences in *tat1* and a 21-bp deletion of redundant sequences in *env* gp41 rapidly become fixed in the evolving virus quasispecies. The version 2 backbone vector eliminates these redundant sequences. (B) Nucleotide and inferred amino acid sequences of the junctional regions of HIV-1 and SIV *tat1* and *env* gp41 version 1 and 2 backbone vectors are shown, highlighting the differences in their designs.

The design strategy for constructing SHIVs is illustrated in Fig. 1A. This construction scheme allowed for the complete extracellular gp140 region of Env plus the transmembrane segment and 9 amino acids (aa) of the cytoplasmic tail (nucleotides 1 to 2153; HXB2 numbering) to be PCR amplified *en bloc* and subcloned into a chimeric T/F SIVmac766-HIV-1 proviral backbone (35). If sequences were available for *vpu* in the source material, then the homologous *vpu-env* gp140 gene segment was amplified and subcloned into the proviral vector, since homologous *vpu-env* sequences could potentially enhance the efficiency of Env translation. Env375 codon substitutions corresponding to Trp, Phe, Tyr, His, or Met were introduced by site-directed mutagenesis into each SHIV construct, which was then prepared as a large-scale DNA stock and sequence confirmed. Genome sequences for all SHIVs were contributed to GenBank (Table 1). For each of the 10 primary HIV-1 Envs, six variants containing the different Env375 alleles were made, bringing the total number of newly constructed SHIVs to 60. In the course of SHIV constructions, we noted that certain aspects of the design scheme were inefficient, especially the requirement for multiple PCR amplifications and ligations (see Materials and Methods). We also found in SHIV-infected RMs that redundant *tat1* and *env* gp41 sequences of 68 and 21 bp in length, respectively, that were generated as a consequence of the original cloning strategy underwent spontaneous deletion (Fig. 1A and Fig. 2) (35, 38). Thus, we modified the SIVmac766 backbone vector and amplification primers to simplify the PCR amplification step and eliminate the redundant sequences (Fig. 1B). We used this new design strategy to reclone SHIV.CH505 in order to perform a head-to-head comparison of viruses expressed from this new vector compared with the original SHIV design, and to clone a new SHIV containing the HIV-1 CH0694 Env. Plasmid DNA for all SHIVs was transfected into 293T cells and virus-containing supernatants were characterized for p27Ag content and infectivity on TZM-bl cells. For all SHIVs, p27Ag concentrations ranged from 200 to 2,000 ng/ml. One nanogram of p27Ag is equivalent to approximately 10^7^ virions, so SHIV titers were estimated to range from 2 × 10^9^ to 2 × 10^10^ virions per ml. We confirmed these titers by quantifying vRNA and assuming two vRNA molecules per virion. Infectivity titers on TZM-bl cells ranged from 2 × 10^5^ to 2 × 10^6^ per ml, corresponding to an IU to particle ratio of approximately 10^−4^. This ratio is typical for 293T-derived HIV-1 and SIV virions (35), and 100-fold lower than for virus stocks propagated in primary rhesus CD4^+^ T cells, where between 1 in 100 and 1 in 50 virions are typically infectious on TZM-bl cells (Table 2) (35).

**TABLE 2:** SHIV challenge stocks expanded in primary rhesus CD4^+^ T cells

Virus stock	vol/ vial (ml)	Date generated	Vials	p27 ng/ml	vRNA copies/ml	Infectivity titer (IU/ml)		IU/particle ratio^*a*^	AID_50_^*b*^
						TZMbl	rhCD4+ T cells		
SHIV.BG505.332N.375Y.dCT(s1^*e*^)	0.75	2/24/16	1,567	154	2,105,956,647	13,437,500	630,957	0.0128	1:3^*c*^, 1:120^*d*^
				270^*g*^	2,263,660,000^*g*^	6,093,000^*g*^	165,000^*g*^	0.0054^*g*^	
SHIV.BG505.332N.375Y.dCT(s2^*f*^)	1.0	1/28/19	2,080	365	4,089,870,000	31,640,000	4,050,000	0.0155
SHIV.CH505.375H.dCT(s1)	0.5	12/22/15	1,194	178	631,771,853	6,797,000	186,209	0.0215	1:2^*c*^, 1:80^*d*^
SHIV.CH505.375H.dCT(s2)	1.0	5/10/17	1,626	190	778,255,000	5,234,400	631,000	0.0135	1:80^*d*^
SHIV.CH848.375H.dCT	1.0	4/28/16	1,355	73	900,447,863	4,421,000	162,000	0.0098
SHIV.191859.375M.dCT	0.25	10/21/14	192	212	3,004,673,792	31,898,389	630,957	0.0212	1:3^*c*^
SHIV.B41.375H.dCT	1.0	10/27/17	1,675	200	1,710,815,153	8,125,000	369,000	0.0095
SHIV.1086.375W.dCT	1.0	2/12/18	2,057	207	502,180,000	146,000	1,870	0.0006
SHIV.CH1012.375Y.dCT	1.0	2/3/20	2,216	552	1,576,865,000	4,687,000	125,000	0.0059
SHIV.Ce1176.375HFW.dCT^*h*^	1.0	2/4/20	2,224	390	1,619,830,000	6,562,000	125,000	0.0081
SHIV.T250-4.375HWY.dCT^*i*^	1.0	2/14/20	1,231	634	1,939,331,667	7,656,000	125,000	0.0079	1:160^*d*^^,^^*j*^

a IU/particle ratio determined on TZM-bl cells assuming two vRNA molecules/virion.

b AID_50_, 50% animal infective does, i.e., the inoculum dose leading to productive clinical infection in 50% of rhesus macaques.

c Intravaginal (IVAG) inoculation route (1 ml of 1:X dilution).

d Intrarectal (IR) inoculation route (1 ml of 1:X dilution).

e First stock expansion.

f Second stock expansion.

g Repeat measurements performed on samples stored for 3 years in vapor phase liquid nitrogen.

h Stock composed of a mixture of Env375 His, Phe, and Trp variants.

i Stock composed of a mixture of Env375 His, Trp and Tyr variants.

j Intrarectal AID_50_ estimate of SHIV.T250-4.375HWY.dCT by D Sok and E Rakasz (unpublished).

**FIG 3:**
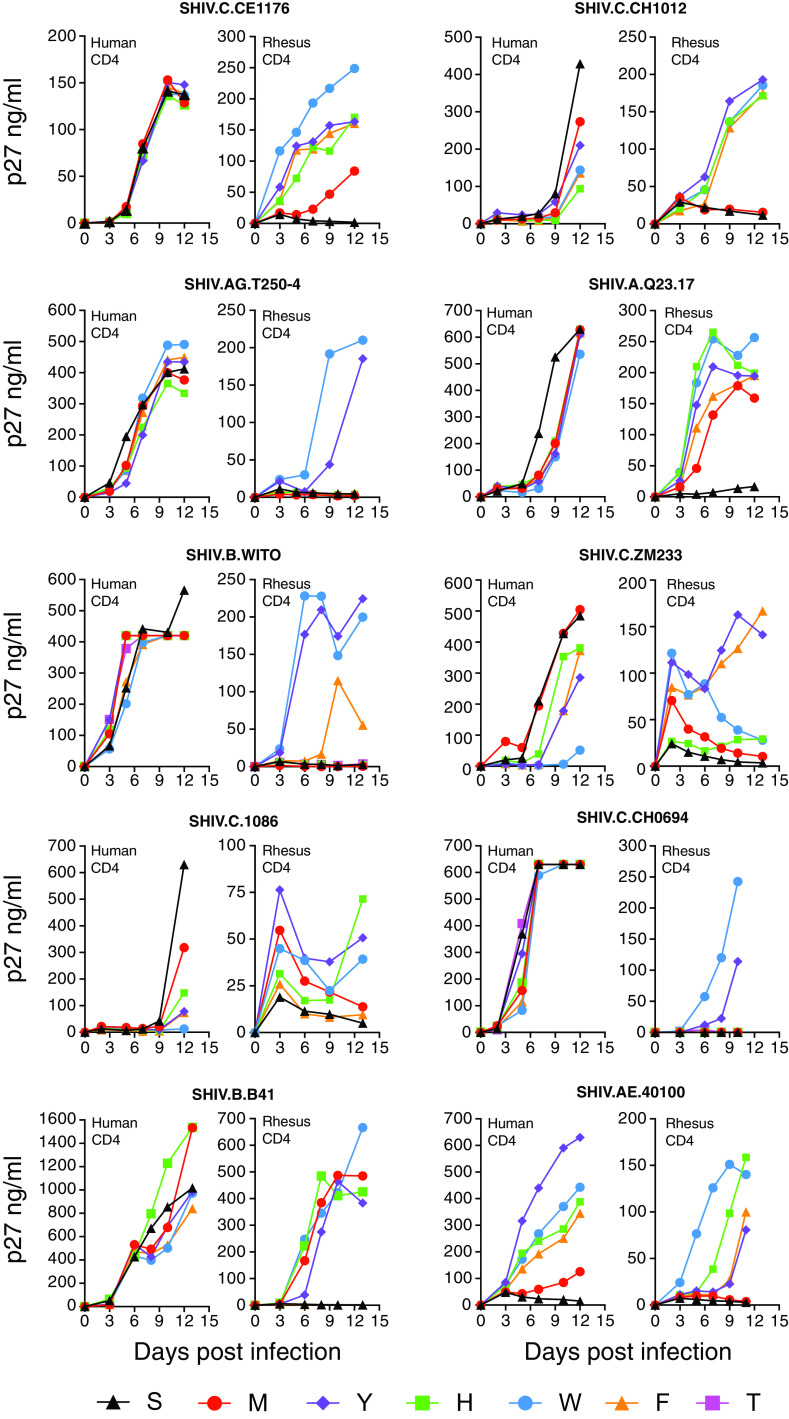
Replication kinetics of SHIVs bearing 10 different HIV-1 Envs with allelic variants at residue Env375 (S, Ser; M, Met; Y, Tyr; H, His; W, Trp; F, Phe; and T, Thr) in cell culture. Primary, activated human and rhesus CD4^+^ T cells were inoculated with 293T-derived SHIVs and culture supernatants were sampled on the days indicated. Panels display results of representative experiments, which were reproduced in large-scale expansions of SHIVs in rhesus CD4^+^ T cells *in vitro* (Table 2) and in RMs *in vivo* (Fig. 4).

For SHIVs bearing the 10 new HIV-1 Envs, we evaluated the replication efficiency of each of them containing six different Env375 residues in primary activated human and rhesus CD4^+^ T cells *in vitro* (Fig. 3). With the exception of SHIV.AE.40100, which naturally contains the positively charged, aromatic residue Env375-His, none of the SHIVs containing wild-type Ser or Thr residues at position Env375 replicated appreciably in rhesus CD4^+^ T cells (Fig. 3). Conversely, all 10 SHIVs with wild-type Env375 residues replicated efficiently in primary activated human CD4^+^ T cells. The latter result—efficient replication of SHIVs containing wild-type 375 alleles in human CD4^+^ T cells—was an expected finding but was nonetheless critical to demonstrate, since it confirmed that the chimeric SHIVs that we made were capable of supporting replication. We next asked if substitution of the wild-type Env375 allele by one or more aliphatic or aromatic residues (Met, Trp, Phe, Tyr, or His) would support SHIV replication in rhesus CD4^+^ T cells. The answer was affirmative for SHIVs expressing each of the 10 HIV-1 Envs (Fig. 3). The differences in virus replication in rhesus CD4^+^ T cells between SHIVs expressing wild-type Env375 residues and those expressing bulky aromatic residues was generally quite large, oftentimes resulting in >100-fold differences in p27Ag concentration in culture supernatants at multiple time points throughout the infection (Fig. 3). Among the six different Env375 alleles that were tested, Env375-Trp most consistently supported SHIV replication in rhesus CD4^+^ T cells; it was effective in all 10 HIV-1 Env backbones. Env375-Tyr was the second most favored residue, followed by Env375-His or -Phe. It is notable that Trp is also the most conserved Env375 allele across the broad evolutionary spectrum of primate lentiviruses excluding humans and great apes (30). These results thus corroborate and extend a substantial body of scientific literature indicating that SHIVs bearing primary (nonadapted) wild-type HIV-1 Envs rarely replicate efficiently in rhesus cells (1–3, 27–29, 35, 38, 65, 66) and that this restriction can be lifted by substituting a single amino acid at position Env375. In our combined studies (this study plus references 35 and 38), we replaced wild-type Env375 residues in 16 primary HIV-1 Envs—15 of which could not support SHIV replication in RMs—and found in all instances that this substitution alone led to efficient SHIV replication in rhesus animals.

**FIG 4:**
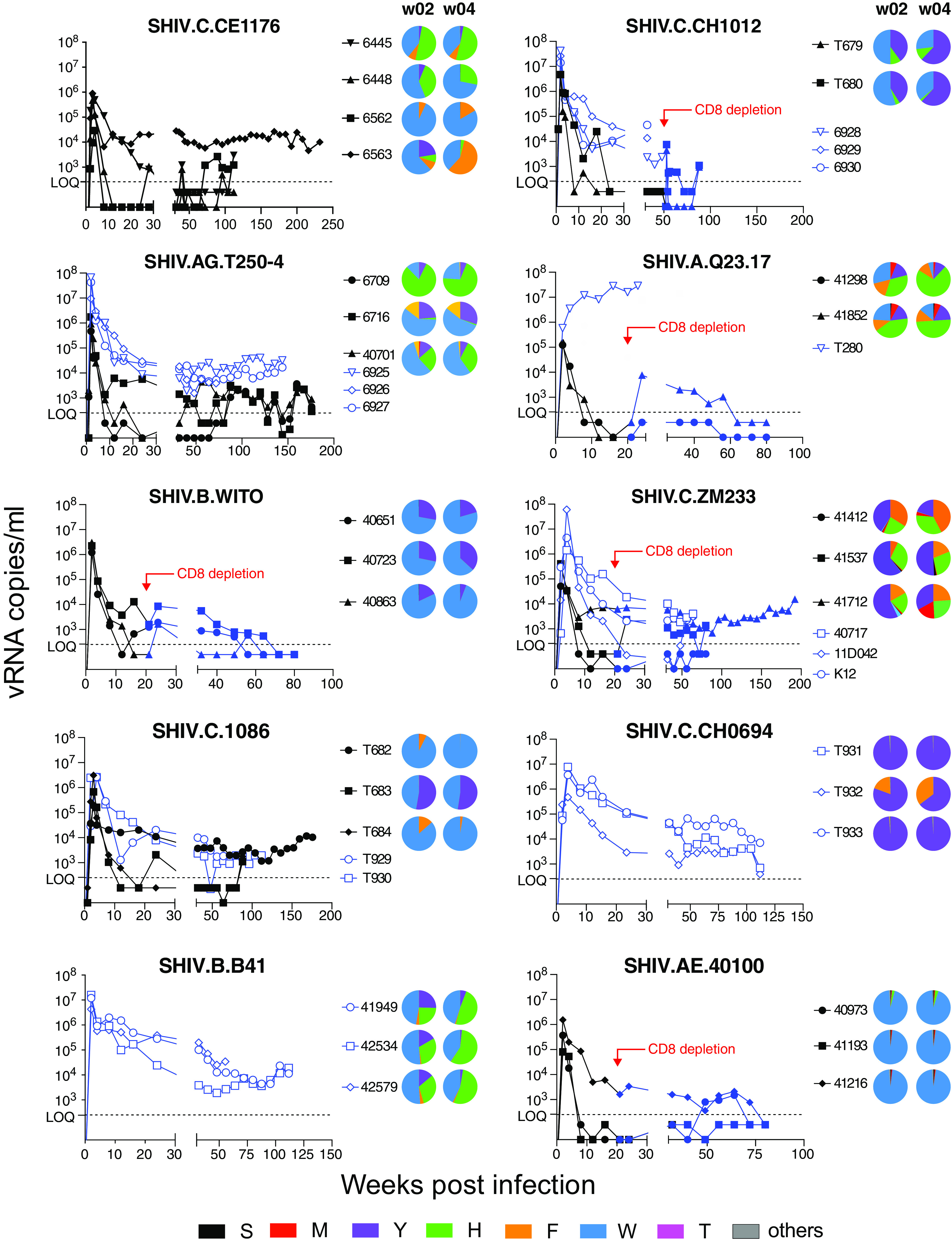
Plasma vRNA kinetics following intravenous inoculation of RMs with SHIVs bearing six Env375 allelic variants. Open symbols denote animals that were treated with anti-CD8 MAb at week 0. Solid black symbols denote animals that were not treated with anti-CD8 MAb at week 0. Some animals were treated with anti-CD8 MAb at time points indicated during the course of SHIV infection and these animals are indicated by a shift in solid symbols from black to blue. Pie diagrams represent >5,000 vRNA/cDNA sequences and indicate the proportions of different Env375 alleles in plasma vRNA at 2 and 4 weeks after SHIV inoculations. Where pie diagrams are not shown, animals were inoculated with SHIVs containing a single Env375 allele (Table 3).

To extend these findings to *in vivo* analyses, we inoculated 41 RMs intravenously in groups of 3 to 6 animals each, with SHIVs containing one of the 10 selected HIV-1 Envs and an equal mixture of the six Env375 alleles based on p27Ag content (Table 3 and Fig. 4). We used this experimental design for two reasons. First, because target cell availability is not limited in the initial 2 weeks of infection, during which time virus titers increase exponentially (67–70), we could use deep sequencing of plasma vRNA/cDNA to directly compare the relative replication rates of the six Env375 allelic variants in an *in vivo* competitive setting. Second, it would be impractical and prohibitively expensive to test 60 SHIVs individually in 60 different monkeys and, even if this could be done, the results would be confounded by monkey-specific variables such as MHC class I and II recognition. Each of the 41 RMs that we inoculated with an SHIV Env375 mixture became productively infected after a single challenge (Fig. 4). In most animals, peak viremia occurred at day 14 post-SHIV inoculation and plasma virus load setpoints were reached 16 to 24 weeks later. Animals treated with anti-CD8 monoclonal antibody (Mab) at the time of SHIV inoculation developed significantly higher peak and setpoint viremia titers compared with untreated animals (*P* < 0.01 for both). A subset of animals was treated with anti-CD8 MAb at setpoint, 20 to 50 weeks after infection, and most of these animals exhibited increases in virus titers. We performed next-generation sequencing (NGS) on plasma samples taken 2 and 4 weeks postinfection to determine the relative replication rates of the different Env375 allelic variants (Fig. 4). We expected that differences in infectivity of the Env375 variants would be reflected in the plasma virus quasispecies by 2 weeks postinoculation, since the combined half-lives of circulating virus and the cells producing it is <1 day (71), resulting in multiple rounds of *de novo* virus infection and replication during this early interval. This was indeed the case. Overall, there was a good correlation between Env375 residues that supported SHIV replication *in vitro* and *in vivo*. For example, in all 10 different Env backgrounds, Env375-Ser failed to support SHIV replication in primary rhesus CD4^+^ T cells *in vitro* (Fig. 3) and the same was true in RMs *in vivo* (Fig. 4). Conversely, Env375-Trp supported SHIV replication in all 10 Env backgrounds *in vitro* and was a predominant allele supporting efficient SHIV replication in 7 of 10 Env backgrounds *in vivo*. There were some differences in Env375 residues that best supported SHIV replication *in vitro* versus *in vivo*. For example, for SHIVs bearing ZM233 and CH0694 Envs, 375-Trp supported efficient virus replication *in vitro* but not *in vivo*, where 375-Tyr was dominant. And the Env375-His allele, which is naturally present in most subtype AE viruses, including the AE.40100 strain, supported efficient SHIV.AE.40100 replication in rhesus CD4^+^ T cells *in vitro* but not *in vivo*. Taken together, the findings indicate that substitution of wild-type Env375 alleles in primary HIV-1 Envs with Trp, Tyr, or His results in SHIV chimeras that replicate efficiently in RMs. However, since it is impossible to predict with certainty which Env375 allele will best support *in vivo* replication of an SHIV bearing any particular HIV-1 Env, an *in vivo* competition experiment similar to that illustrated in Fig. 4 must be conducted.

**TABLE 3:** Characteristics of SHIV inocula and clinical outcomes of 41 rhesus macaques

SHIV strain	Subtype	Wild-type 375 residue	Animal ID	375 variant	No. of variants	Stock derivation	Dosage P27 ng^*a*^	Route^*b*^	CD8 depletion	Peak VL^*c*^	Setpoint VL^*c*^	Preferred 375 residues^*d*^	Clinical AIDS
CE1176	C	375-Ser	6445	S, M, H, Y, F, W	6	293T	300	i.v.	No	673,106	1,016	H, W	No
			6448	S, M, H, Y, F, W	6	293T	300	i.v.	No	482,934	<250	W, H	No
			6562	S, M, H, Y, F, W	6	293T	300	i.v.	No	29,252	<250	W, F	No
			6563	S, M, H, Y, F, W	6	293T	300	i.v.	No	894,432	20,608	F, W	No
CH1012	C	375-Ser	T679	S, M, H, Y, F, W	6	293T	300	i.v.	No	4,814,098	<250	Y, W, H	No
			T680	S, M, H, Y, F, W	6	293T	300	i.v.	No	4,759,788	<250	Y, W	No
			6928	Y	6	293T	50	i.v.	Yes	42,350,830	8,830	No
			6929	Y	6	293T	50	i.v.	Yes	25,654,060	39,992	Yes
			6930	Y	6	293T	50	i.v.	Yes	14,495,480	10,656	Yes
T250-4	AG	375-Ser	6709	S, M, H, Y, F, W	6	293T	300	i.v.	No	471,828	<250	H, W	No
			6716	S, M, H, Y, F, W	6	293T	300	i.v.	No	1,744,646	5,708	W, Y, F	No
			40701	S, M, H, Y, F, W	6	293T	300	i.v.	No	932,684	0	W, H	No
			6925	Y, H, W, F	1	293T	200	i.v.	Yes	67,161,230	8,900	H	No
			6926	Y, H, W, F	1	293T	200	i.v.	Yes	9,600,904	29,196	H	Yes
			6927	Y, H, W, F	1	293T	200	i.v.	Yes	42,558,680	23,966	H	No
Q23.17	A	41298	S, M, H, Y, F, W	6	293T	300	i.v.	No	122,560	<250	H, Y, F, W	No
		375-Ser	41852	S, M, H, Y, F, W	6	293T	300	i.v.	No	173,420	7,680	H, Y, F, W	No
		T280	H	1	293T	50	i.v.	Yes	29,118,930	29,000,000	Yes
WITO4160	B	375-Thr	40651	T, S, M, H, Y, F, W	7	293T	350	i.v.	No	1,222,790	1,922	W, Y	No
			40723	T, S, M, H, Y, F, W	7	293T	350	i.v.	No	2,226,030	8,564	W, Y	No
			40863	T, S, M, H, Y, F, W	7	293T	350	i.v.	No	3,044,620	1,648	W, Y	No
ZM233	C	375-Ser	41412	S, M, H, Y, F, W	6	293T	300	i.v.	No	51,052	<250	F, H, Y	No
			41537	S, M, H, Y, F, W	6	293T	300	i.v.	No	408,840	2,390	Y, H, F	No
			41712	S, M, H, Y, F, W	6	293T	300	i.v.	No	364,598	7,068	Y, F, H, M	No
			40717	Y	1	293T	50	i.v.	Yes	1,432,284	18,936	No
			11D042	Y	1	293T	50	i.v.	Yes	61,300,980	88	No
			K12	Y	1	293T	50	i.v.	Yes	4,445,834	2,072	No
1086	C	375-Ser	T682	S, M, H, Y, F, W	6	293T	300	i.v.	No	190,208	11,062	W	Yes
			T683	S, M, H, Y, F, W	6	293T	300	i.v.	No	682,242	2,090	W, Y	No
			T684	S, M, H, Y, F, W	6	293T	300	i.v.	No	3,144,292	<250	W	No
			T929	W	1	293T	50	i.v.	Yes	2,679,424	20,138	No
			T930	W	1	293T	50	i.v.	Yes	2,648,942	7,944	No
CH0694	C	375-Thr	T931	T, S, M, H, Y, F, W	7	293T	350	i.v.	Yes	7,778,446	106,716	Y	No
			T932	T, S, M, H, Y, F, W	7	293T	350	i.v.	Yes	472,192	2,928	Y, F	No
			T933	T, S, M, H, Y, F, W	7	293T	350	i.v.	Yes	3,718,208	119,506	Y	No
B41	B	375-Ser	41949	S, M, H, Y, F, W	6	293T	300	i.v.	No	11,980,270	285,856	H, W, Y	No
			42534	S, M, H, Y, F, W	6	293T	300	i.v.	No	16,044,860	24,314	H, W	No
			42579	S, M, H, Y, F, W	6	293T	300	i.v.	No	4,332,634	387,742	H, W	Yes
RV217.40100	AE	375-His	40973	S, M, H, Y, F, W	6	293T	300	i.v.	No	370,894	0	W	No
			41193	S, M, H, Y, F, W	6	293T	300	i.v.	No	81,162	0	W	No
			41216	S, M, H, Y, F, W	6	293T	300	i.v.	No	1,555,258	3,380	W	No

a Inocula consisted of 50 ng p27Ag equivalent of each Env375 SHIV variant.

b i.v., intravenous bolus by slow push.

c VL, virus load (vRNA molecules/milliliter of plasma).

d Preferred Env375 allelic variants in plasma (H, His; W, Trp; F, Phe; Y, Tyr; M, Met).

**FIG 5:**
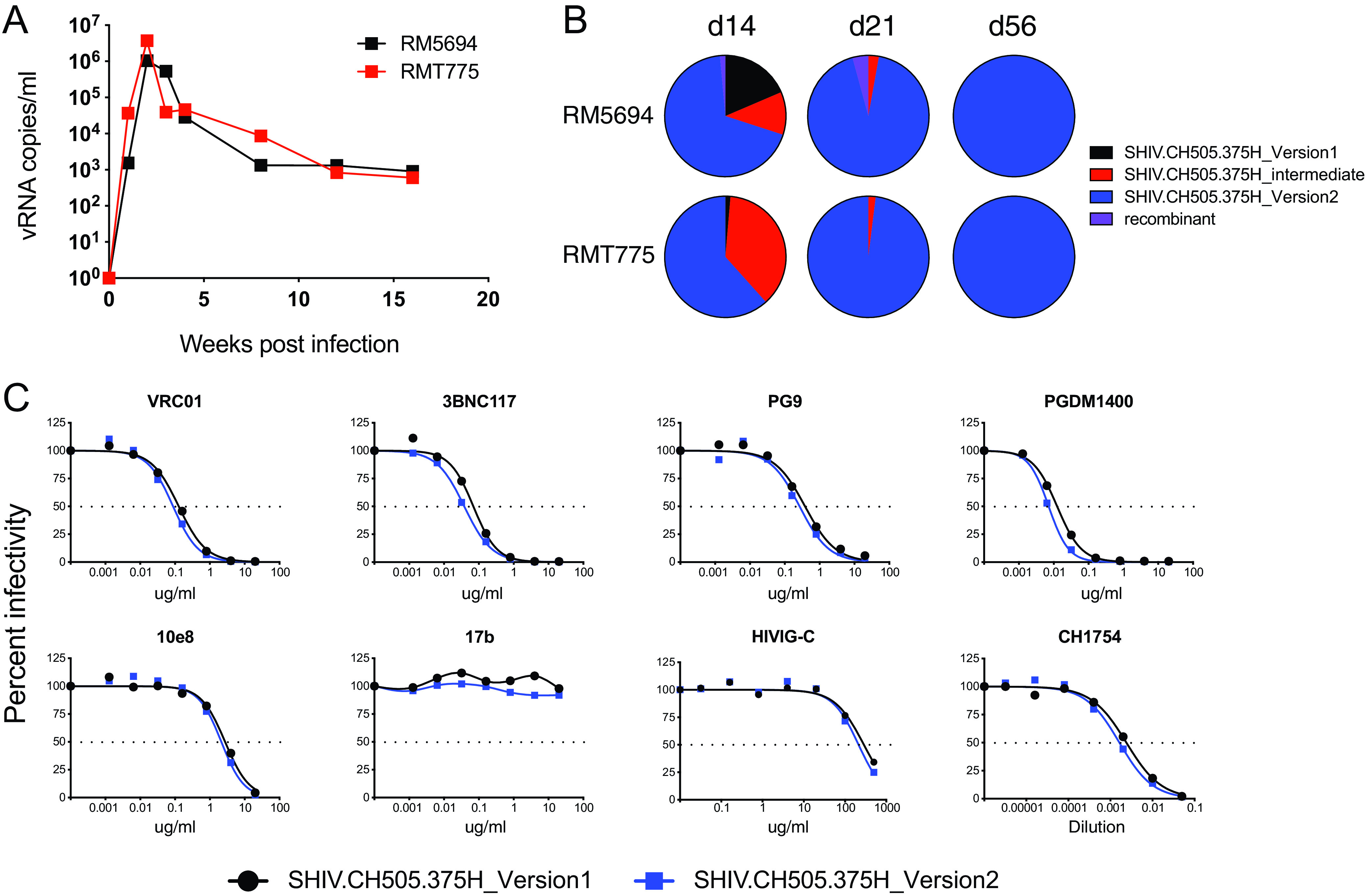
Comparison of replication efficiency, antigenicity, and neutralization sensitivity of SHIV.C.CH505.375H derived from the first-generation design scheme (GenBank accession no. KU958487) and the second-generation design scheme (GenBank accession no. MW410742). Equal proportions of SHIV.CH505.375H version 1, version 2, and an “intermediate version” where only the 5′ *tat* redundancy was eliminated, were inoculated intravenously into two RMs. (A and B) Replication kinetics were determined by plasma vRNA quantification (A) and relative proportions of the three variants were determined by single genome sequencing (B). (C) The neutralization of version 1 and 2 SHIVs by prototypic human bNAb MAbs, the CD4-induced bridging sheet targeted MAb 17b, and polyclonal anti-HIV-1 IgG (HIVIG-C) and serum (CH1754).

We also compared the relative replication efficiency of SHIV.CH505.375H generated by the first- and second-generation construction strategies (Fig. 5). We showed previously that, in animals infected by viruses produced from the first-generation design, redundant *tat1* and *env* gp41 sequences (68 and 21 bp, respectively) were spontaneously deleted following prolonged *in vivo* replication (Fig. 2) (35, 38). This suggested a fitness disadvantage for viruses containing the redundant sequences, leading us to hypothesize that animals infected by an equal mixture of the viruses derived from the two designs would show preferential replication by viruses lacking the redundant sequences. This was indeed the case (Fig. 5A and B). At 3 weeks postinfection, viruses lacking the redundant sequences comprised >95% of the plasma virus quasispecies, and by week 8, they comprised >99% of plasma virus.

**FIG 6:**
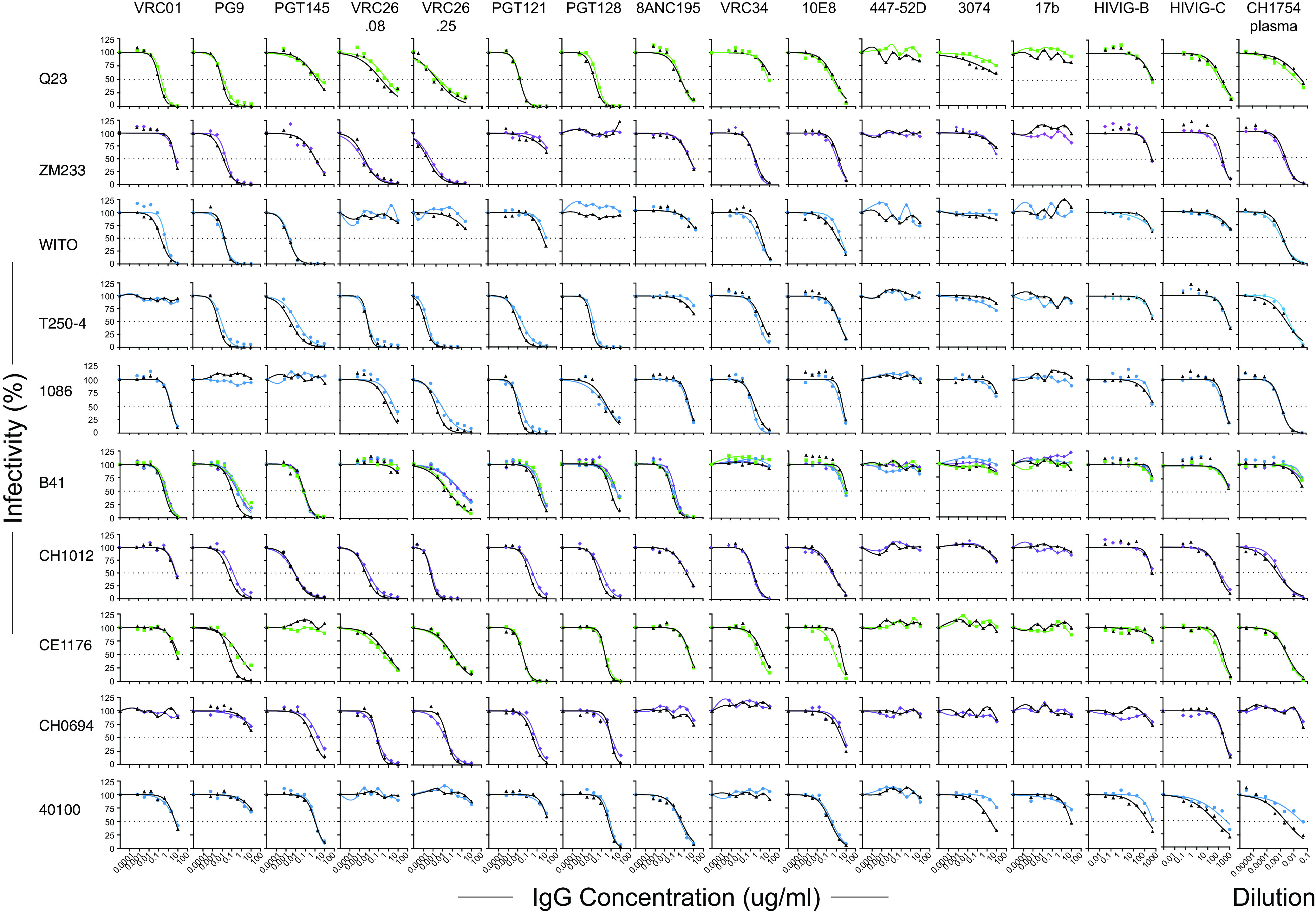
Neutralization sensitivity of SHIVs bearing wild-type Env375 residues (black symbols) or alternate Env375 residues preferred for replication in RMs (Trp, blue; His, green; Tyr, purple). Human 293T cell-derived virus was assayed for entry into TZM-bl cells in the presence or absence of anti-HIV-1 MAbs, polyclonal human anti-HIV IgG preparations (HIVIG-B, -C), or plasma from a human subject CH1754 chronically infected by HIV-1 (103). The *x* axis depicts antibody concentration (μg/ml) except for CH1754 plasma, which is depicted as a percentage dilution.

**FIG 7:**
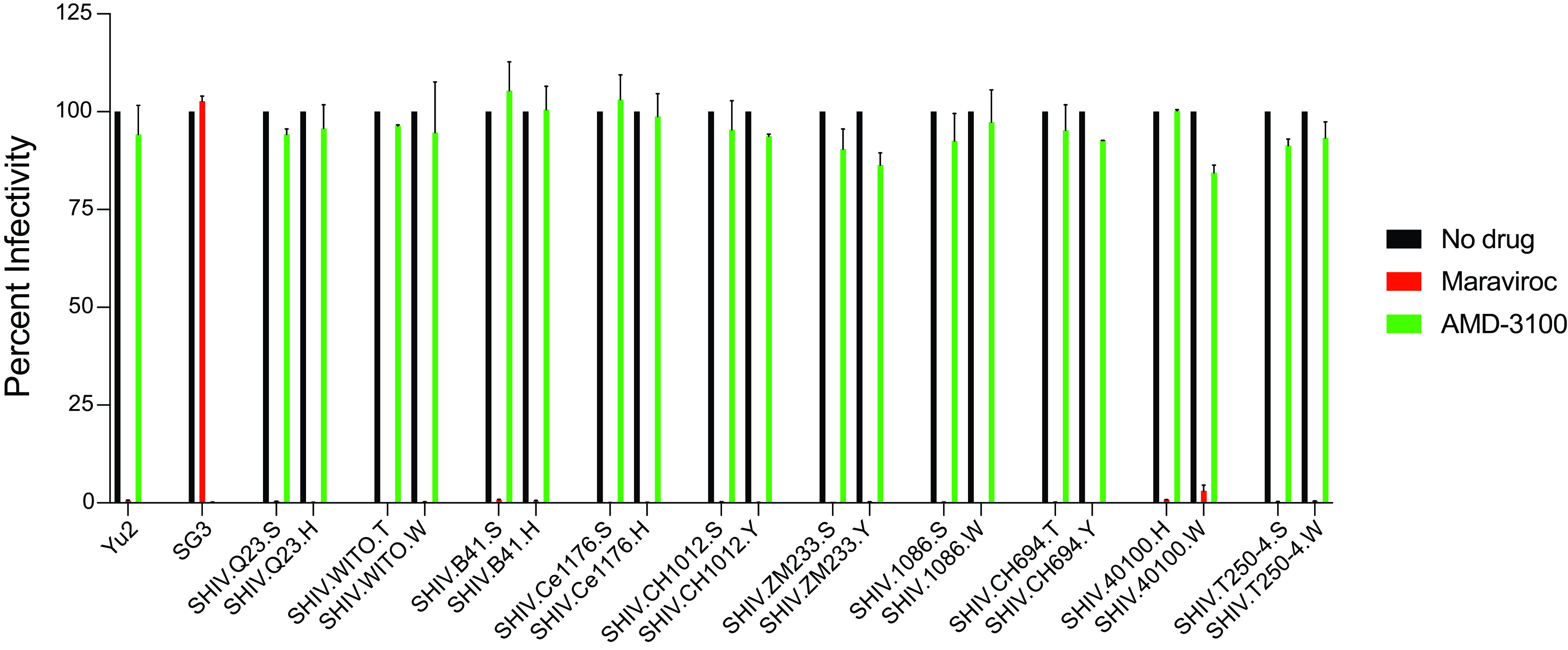
Inhibition of SHIV entry into TZM-bl cells by the selective CCR5 and CXCR4 coreceptor inhibitors Maraviroc and AMD-3100, respectively. HIV-1 YU2 Env utilizes CCR5 exclusively for cell entry and HIV-1 SG3 Env utilizes CXCR4 exclusively, as shown. Entry of the 10 SHIVs bearing wild-type or rhesus-preferred Env375 alleles was inhibited >99% by Maraviroc but minimally or not at all by AMD-3100, indicating dependence on CCR5 for cell entry.

To be a relevant model for HIV-1 vaccine studies, SHIV Envs should exhibit clinically relevant antigenic profiles, neutralization sensitivity phenotypes, and coreceptor usage indistinguishable from the primary HIV-1 Envs from which they were derived. We evaluated the neutralization sensitivity patterns of Envs expressing the wild-type Env375 allele compared with Envs expressing one or more of the alternative Env375 alleles that were found to support replication in rhesus CD4^+^ T cells *in vitro* (Fig. 3) and in RMs *in vivo* (Fig. 4). SHIVs were analyzed using polyclonal anti-HIV-1 sera and a battery of monoclonal antibodies (MAbs) that bind canonical bNAb epitopes, linear V3 epitopes, or CD4-induced (CD4i) epitopes (Fig. 6). Linear V3 and CD4i epitopes are generally concealed on native Env trimers from primary viruses (40, 72–74), and thus MAbs targeting these epitopes typically fail to neutralize primary virus strains. Conversely, neutralization by linear V3 or CD4i MAbs is generally an indication of a nonnative “open” trimer structure, typical of laboratory-adapted viruses. In none of the 10 primary Env backbones that we tested did Env375 substitutions result in neutralization by linear V3 or CD4i MAbs (Fig. 6). Nor did Env375 mutations alter the neutralization sensitivity of these Envs to HIVIG B, HIVIG C, or a high titer, broadly neutralizing HIV-1-infected patient plasma specimen CH1754. These results suggest that the Envs bearing residue 375 substitutions retained their native or near-native conformation. These Envs also retained their antigenicity with respect to bNAb epitope presentation, since MAbs targeting CD4bs, V2 apex, V3 high mannose patch, and MPER sites exhibited similar neutralization patterns against wild-type and Env375 substituted variants. It is notable that the contours of the neutralization curves, the 50% inhibitory concentration (IC_50_), IC_80_, and IC_90_ values, and the steep sigmoidal inflections, were generally indistinguishable between wild-type Envs and Envs bearing residue 375 substitutions. SHIV.Q23.17 demonstrated neutralization sensitivity patterns to the bNAb MAbs, the three polyclonal anti-HIV IgG and plasma reagents, and the MAbs targeting linear V3 or CD4i epitopes that were similar to the other nine SHIVs, thus supporting a tier 2 status for this virus. We also tested SHIV.CH505.375H derived by first- and second-generation design schemes for sensitivity to HIV-1 bNAbs, the CD4i MAb 17b, HIVIG-C, and the anti-HIV-1 broadly neutralizing polyclonal plasma CH1754; the two virus preparations showed indistinguishable neutralization sensitivity patterns (Fig. 5C). Finally, the SHIVs containing the 10 new HIV-1 Envs were tested for coreceptor usage by analyzing their sensitivity to AMD-3100 (a CXCR4 inhibitor) and Maraviroc (a CCR5 inhibitor). Maraviroc, but not AMD-3100, inhibited the entry of all 10 SHIVs in the TZM-bl entry assay (Fig. 7), thus demonstrating CCR5-dependent entry. Altogether, the results indicate that Env375 substitutions did not appreciably alter the antigenicity, tier 2 neutralization sensitivity, or CCR5 tropism of any of the 10 SHIVs.

**FIG 8:**
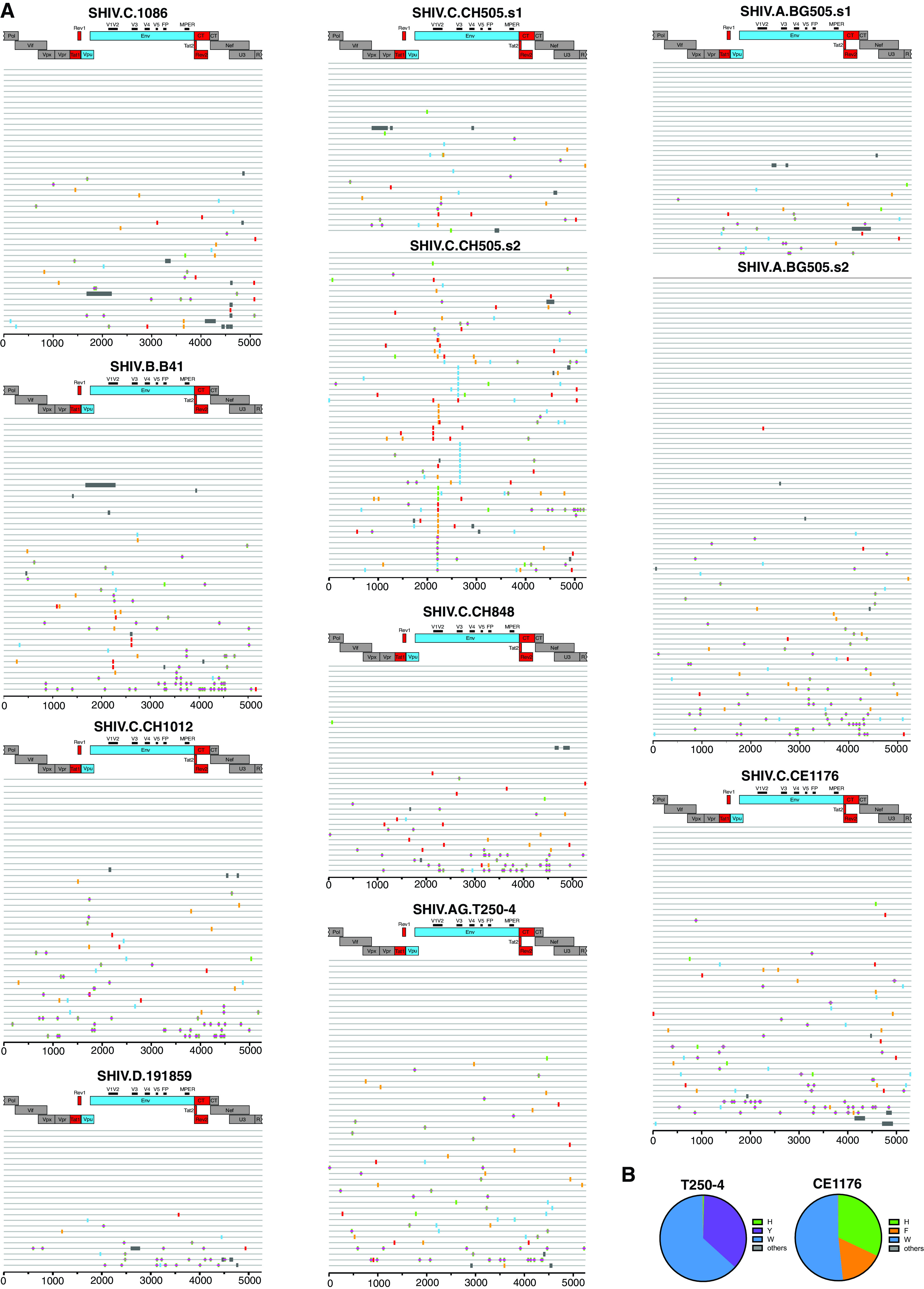
(A) Pixel plots (https://www.hiv.lanl.gov/content/sequence/pixel/pixel.html) of single genome sequences of 3′ half genomes of rhesus CD4^+^ T cell-grown SHIV challenge stocks. Tic marks indicate nucleotide differences from the SHIV molecular clones (T, red; G, yellow; C, blue; A, green; APOBEC site, green+pink; indels, gray). GenBank accession numbers of the SHIV challenge stock sequences are MW508063 to MW508110 (SHIV.C.1086); MW508111 to MW508159 (SHIV.B.B41); MW508160 to MW508202 (SHIV.C.CH1012); MW508203 to MW508226 (SHIV.D.191859); MW507934 to MW507964 (SHIV.C.CH505.s1); MW507965 to MW508022 (SHIV.C.CH505.s2); MW508023 to MW508062 (SHIV.C.CH848); MW508227 to MW508279 (SHIV.AG.T250-4); MW484951 to MW484987 (SHIV.A.BG505.s1); MW507843 to MW507933 (SHIV.A.BG505.s2); and MW508280 to MW508333 (SHIV.C.CE1176). (B) Pie diagrams showing the relative proportions of different Env375 alleles in SHIV.C.CE1176 and SHIV.AG.T250-4 challenge stocks (see the text for explanation).

SHIVs intended for use as challenge strains in preclinical vaccine trials can be generated from 293T cells by transfection of proviral DNA or by virus passage and expansion in primary human or rhesus CD4^+^ T cells. Each approach has potential advantages and disadvantages (3, 75). We chose to prepare challenge stocks by infecting primary, activated rhesus CD4^+^ T cells with molecularly cloned virus derived from 293T cell transfections and then expanding the virus as rapidly as possible, so as to minimize chances for culture adaptation. By this means, we could ensure that the viral envelopes of challenge stocks contained exclusively rhesus (not human) membrane-associated proteins and that glycosylation patterns would be of rhesus (not human) origin. We selected nine SHIV strains for large scale expansion in rhesus cells and these are listed in Table 2. These SHIVs were chosen to be representative of global HIV-1 diversity, including subtypes A, B, C, D, and AG, and to include SHIVs bearing BG505.N332, CH505, and 1086 Envs, which correspond to vaccine candidates in current or recent human clinical trials. Our aim was to generate large numbers of identical replicates of each SHIV stock (>1,000 vials per SHIV), which could then be characterized biophysically for genetic composition, particle content, infectivity, antigenicity, and neutralization sensitivity and cryopreserved in vapor phase liquid nitrogen (<160°C) for subsequent distribution to the wider scientific community as validated, standardized SHIV challenge stocks. Thus, we inoculated cultures of 100 to 200 million primary, activated, rhesus CD4^+^ cells pooled from three naive Indian RMs at a multiplicity of infection (MOI) of approximately 0.01 with genetically homogeneous, sequence-confirmed, 293T transfection-derived virus stocks. For SHIV.CE1176, we infected primary rhesus cells with an equal mixture of Env375-His, Phe, and Trp alleles, and for SHIV.T250-4 we infected cells with an equal mixture of Env375-His, Tyr, and Trp alleles, because no one of these allelic variants had shown preferential replication in all animals tested (Fig. 4). The other SHIV challenge stocks were generated with single Env375 alleles (Table 2). On days 7 and 14 post-SHIV inoculation, we added new medium and approximately 100 to 200 million fresh, uninfected rhesus CD4^+^ T cells from three different naive RMs so as to expand cell numbers and culture volumes while maintaining cell concentrations between 1 and 2 million per milliliter. Beginning on day ∼10 post-SHIV inoculation, we collected the total volume of culture supernatant and replaced it with a greater volume of fresh medium. This complete medium collection and replacement was then repeated every 4 days through day 21. By this means, we could collect as much as 2.5 liters of culture medium containing each SHIV over a period of approximately 21 days. Each supernatant collection was centrifuged twice at 2,500 rpm for 15 min to remove any residual cells or cell debris and then immediately frozen in bulk at −80°C. Supernatants were not filtered, so as to retain the highest possible infectivity titers. Thus, most of the virus that was collected and frozen during the 18 to 21 day culture period was <4 days old and underwent only one freeze-thaw cycle prior to final vialing. After all supernatant collections had been made, they were thawed at room temperature, combined in a sterile 3-liter flask to ensure complete mixing, and then aliquoted into as many as 2,500 cryovials, generally at 1 ml per vial. The vials were then transferred to vapor phase liquid nitrogen for long-term storage. By this means, we could ensure that all vials for any particular SHIV challenge stock were virtually identical in their contents. Between 192 and 2,224 vials per SHIV, each containing between 0.25 and 1.0 ml of challenge stock, were cryopreserved (Table 2). Validation analyses were done on thawed cryovial samples to ensure results would be representative of all cryopreserved samples. Challenge stocks were free of bacterial or fungal contamination based on culture on thioglycolate broth. The p27Ag concentrations ranged from 73 to 634 ng/ml and vRNA concentrations ranged from 5.0 × 10^8^ to 4.1 × 10^9^ vRNA/ml. Infectivity was tested on TZM-bl cells, where it ranged from 1.5 × 10^5^ to 3.2 × 10^7^ IU/ml, and on primary rhesus CD4^+^ T cells, where it ranged between 1.9 × 10^3^ to 4.1 × 10^6^ IU/ml. The genetic composition of the SHIV challenge stocks was analyzed by single genome sequencing of 3′ half-genomes to validate the authenticity of each stock and to determine if there was evidence of selection *in vitro* (Fig. 8A). Stocks of SHIV.CE1176 and SHIV.T250-4 were sequenced by Illumina deep sequencing to determine the relative proportion of the different Env375 alleles in the final challenge stocks (Fig. 8B). Envelope sequence mean and maximum diversity averaged 0.05% (range 0.03 to 0.13%) and 0.30% (range 0.15 to 0.42%), respectively, in the nine challenge stocks. Mutations across the complete gp160 were essentially random in all challenge stocks except in a secondary expansion of SHIV.CH505. This challenge stock was prepared by infecting naive rhesus CD4^+^ T cells with virus from the first expansion of SHIV.CH505 in an attempt to expand sequence diversity and increase infectivity titers. Maximum sequence diversity and maximum sequence divergence from the T/F sequence were 0.35% and 0.29% for stock number 2 compared with 0.15% and 0.08%, respectively, for stock number 1. The p27Ag and vRNA concentrations and infectivity titers on TZM-bl cells were similar for stocks number 1 and number 2 and infectivity titers on primary rhesus CD4^+^ T cells were about 3-fold higher for stock number 2 compared with stock number 1.

**FIG 9:**
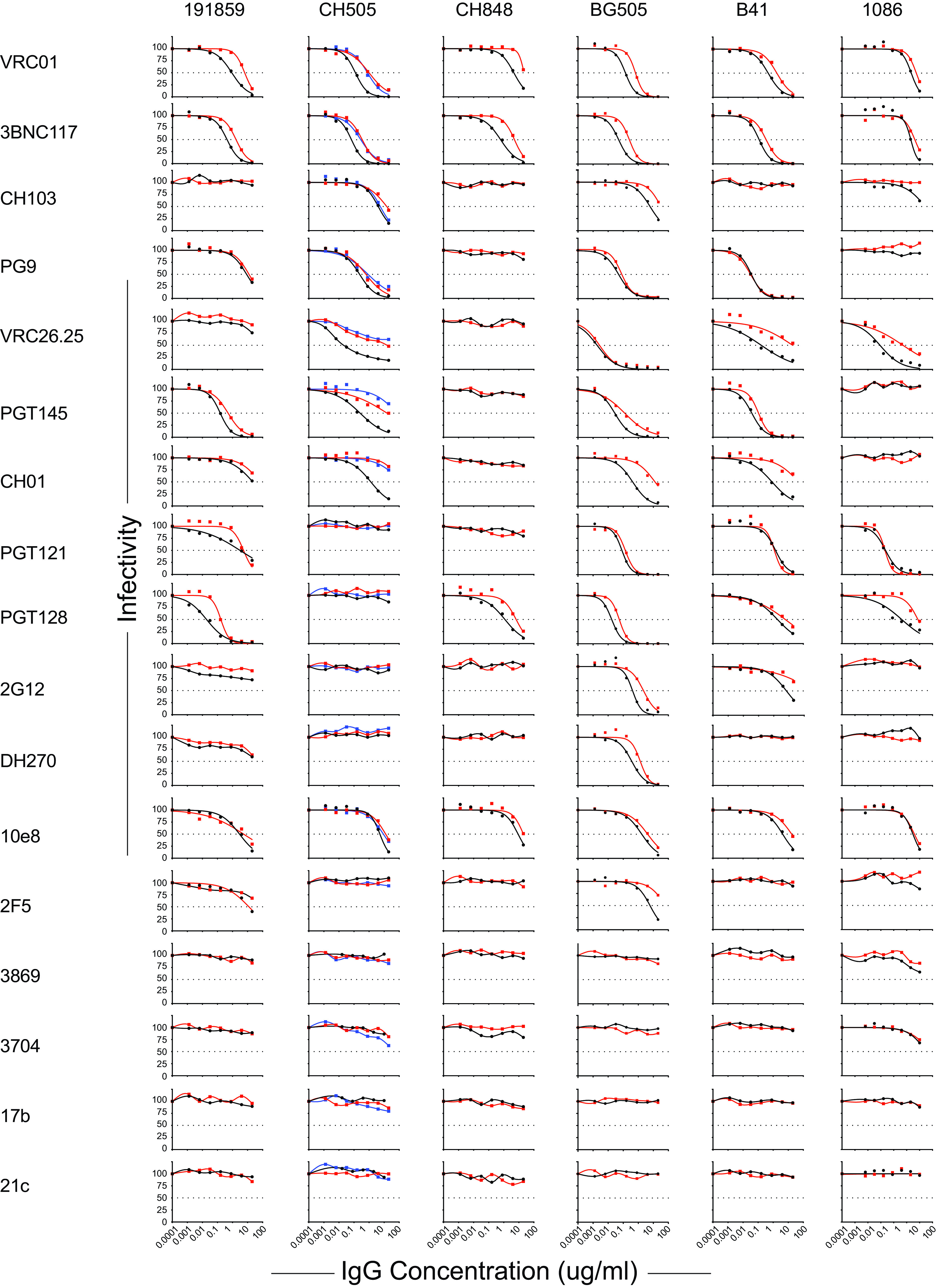
Neutralization sensitivity of SHIVs bearing rhesus-preferred Env375 residues and generated in either 293T cells (black symbols) or primary rhesus CD4^+^ T cells (red symbols). For SHIV.C.CH505, the rhesus CD4^+^ T cell-derived stock 1 is depicted by red symbols and stock 2 by blue symbols.

HIV-1 strains produced in primary human CD4^+^ T cells, compared with the same viruses produced in 293T cells, have been reported to exhibit variably greater resistance to neutralizing antibodies (76, 77). These differences have been attributed to differences in Env content, cell adhesion molecules, surface glycan composition, or other factors (75). We tested six SHIVs (BG505, CH505, CH848, B41, D.191859 and 1086) produced in primary rhesus CD4^+^ T cells and in 293T cells for sensitivity to 17 neutralizing MAbs that targeted CD4bs, V3 glycan, V2 apex, MPER, surface glycan, CD4i, or linear V3 epitopes (Fig. 9). None of the viruses, regardless of cell derivation, were sensitive to the four MAbs that targeted CD4i or linear V3 epitopes, indicating that they retained a native-like closed Env trimer regardless of the cell of origin. SHIVs produced in 293T cells and primary rhesus cells also exhibited similar overall patterns of sensitivity to the other 15 MAbs, in that if an SHIV was sensitive (or resistant) to neutralization by a particular MAb, then this was true regardless of its cell of origin. However, as reported for HIV-1 strains, we observed enhanced resistance to some MAbs by some SHIVs grown in primary rhesus cells compared with 293T cells. This difference was 2- to 5-fold for all six SHIV strains when exposed to VRC01 and 3BNC117 and as much as 25-fold for certain other virus-antibody combinations, such as BG505-CH01, CH505-CH01, CH505-PGT145, CH505-VRC26.25, B41-CH01, B41-VRC26.25, 1086-PGT128, and 1086-VRC26.25 (Fig. 9).

**FIG 10:**
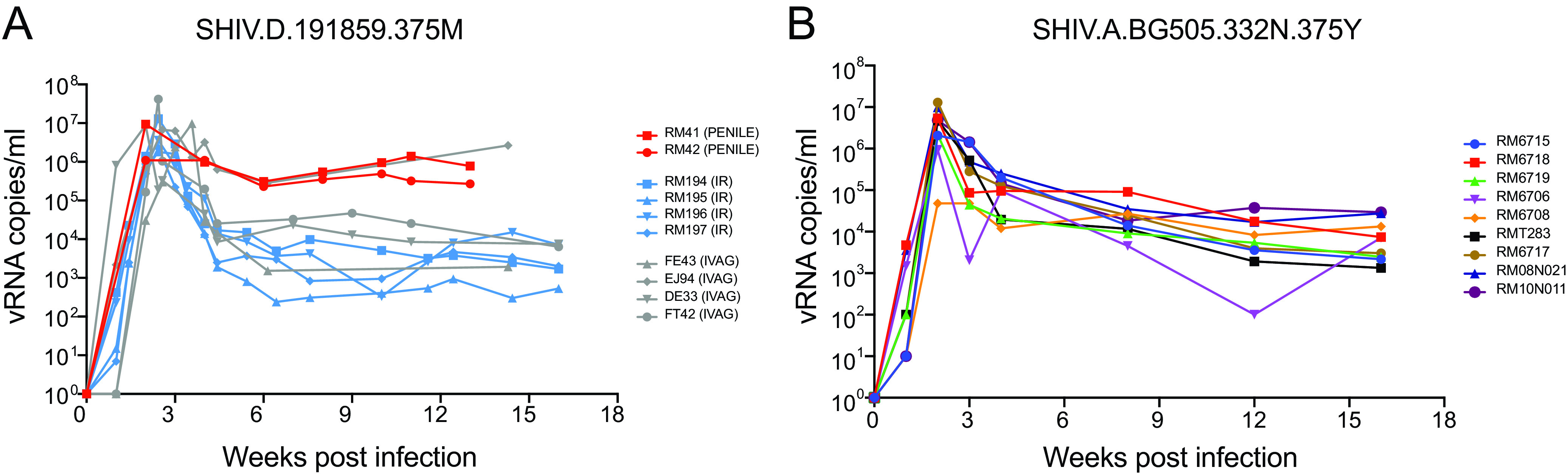
Plasma viral load kinetics following atraumatic penile inoculation of SHIV.D.191859 (A) and atraumatic intrarectal inoculation of SHIV.A.BG505.N332.375Y (B). Two RMs (RM41 and RM42) were inoculated atraumatically a single time with 0.25 ml of undiluted SHIV.D.191859 stock into the sulcus between penile glans and foreskin. This resulted in productive clinical infection in both animals, as indicated by plasma viremia 1 week later. Plasma viral load kinetics through 13 weeks of follow-up were comparable to SHIV.D.191859 plasma virus loads resulting from other routes of transmission where data were reported earlier (35, 101) and similar to plasma viremia of SHIV.A.BG505.N332.375Y following low-dose intrarectal challenge (B). Of note, none of the 19 RMs depicted in panels A and B had been treated with anti-CD8 MAb, thus demonstrating consistent replication kinetics by different SHIVs administered by different mucosal inoculation routes.

The properties of rhesus CD4^+^ T cell-grown SHIV challenge stocks as summarized in Table 2, especially their consistently high virus titers and infectivity measurements, suggested that these virus strains might be suitable for mucosal transmission studies and to assess the preclinical efficacy of actively induced or passively administered bNAbs. Nearly all natural routes of HIV-1 acquisition result from transmission across mucosal surfaces, the exceptions being intrauterine and intravenous infections. Previously, we showed that SHIVs BG505, CH505, and D.191859 can be transmitted efficiently across rectal, vaginal, and oral mucosae (17, 35), resulting in productive clinical infection with virus replication kinetics and plasma virus titers indistinguishable from human infections by HIV-1 (69, 70). Penile acquisition is another important route of HIV-1 transmission in humans (78), and Fig. 10A shows that SHIV.D.191859 can be transmitted by atraumatic inoculation of foreskin and glans. Peak viremia occurred at approximately 2 weeks postchallenge and plasma virus load setpoint was reached by 6 weeks. Setpoint viremia persisted at 50,000 to 200,000 vRNA molecules per milliliter through 16 weeks of follow-up, when the experiment was terminated per protocol. These kinetics of SHIV.D.191859 replication post penile transmission were similar to plasma virus load kinetics of the same SHIV strain transmitted by intrarectal, intravaginal, and intravenous routes (Fig. 10A).

Lastly, we performed a low-dose, repetitive challenge rectal titration of SHIV.BG505.N332 in 12 naive RMs to estimate the 50% animal infectious doses (AID_50_) of the challenge stock and to assess plasma viral load kinetics following IR infection. Three of four inoculations at a dose of 1:20 (1 ml), 3 of 4 inoculations at a dose of 1:100 (1 ml), and 3 of 9 inoculations at a dose of 1:160 (1 ml) resulted in productive clinical infection. Acute and early SHIV.BG505.N332 replication kinetics (Fig. 10B) were similar to mucosal infection by SHIV.D.191859 (Fig. 10A) and also similar to the 10 SHIVs illustrated in Fig. 4 that were infected intravenously. Although our intrarectal AID_50_ titration experiment for SHIV.BG505.N332 involved a small number of animals (*n* = 12) and was subject to stochastic effects related to intrarectal virus inoculation, we could nonetheless estimate the AID_50_ of this stock to be approximately 1:120 (1 ml) for atraumatic IR challenge. This result was corroborated in the control (sham-treated) arm of a preclinical trial assessing the protective efficacy of BG505 SOSIP vaccine-elicited neutralizing antibodies against a homologous SHIV.BG505.N332 challenge (7).

## DISCUSSION

In recent years, there have been notable advances in HIV prevention and cure research (79–83), yet the goals of effective vaccination and cure—even a “functional” cure—seem far in the distance. Increasingly, experimental medicine trials in humans have been pursued as a strategy to accelerate translational research (82) but, at the same time, there remain untapped opportunities and needs for animal models to complement and synergize with human studies to hasten progress. Different scientific questions demand different model systems, ranging from transgenic or humanized mice to outbred small and large animals. Aside from the great apes, which are endangered and thus precluded from laboratory investigation, the rhesus macaque monkey (Macaca mulatta) is most similar to humans in its immune repertoire (84, 85). For HIV-related investigations in primates, two classes of viruses are broadly used: (i) simian immunodeficiency viruses (SIV) and (ii) chimeric SHIVs that express HIV-1 Envs within an SIV background (3). The present study (i) adds 10 new SHIVs to the research portfolio of HIV investigators; (ii) characterizes key biological properties of these SHIVs that are relevant to virus transmission, prevention, immunopathogenesis, and cure research; and (iii) describes a new SHIV design strategy and cloning vector that can facilitate future SHIV constructions.

The HIV-1 Env glycoprotein is critical to virus transmission, persistence, and pathogenesis since it conveys the essential functions of receptor and coreceptor binding and membrane fusion. At the same time, Env is the target of an array of neutralizing antibodies and cytotoxic T cells that cause it to evolve continuously in order to escape recognition that would otherwise lead to virus elimination (38, 86, 87). Env accomplishes the latter by means of highly evolved properties, including occlusion of trimer-interface epitopes (88), epitope variation (89), conformational masking (90), and glycan shielding (91). Although HIV-1 Env is notorious for its variability and global diversity (www.hiv.lanl.gov), it is nonetheless constrained in its potential for immediate or near-term evolution due to the myriad of essential biological functions encoded in its sequence (38, 92–94). These constraints can be lifted, however, by prolonged *in vitro* cultivation (66) or extensive passage in unnatural animal hosts (1–3, 22). The implication of these observations is that the most relevant HIV-1 Envs (95) for studies of vaccine-elicited protection, passively acquired antibody protection, or curative intervention are primary or T/F Envs from viruses that are responsible for clinical transmission and the establishment of persistent infection in humans (7–10, 96). T/F Envs express the precise primary, secondary, tertiary, and quaternary protein structures that are essential for transmission and T/F Envs are the ones that a vaccine-elicited bNAb response must recognize if it is to be protective (38, 73, 82). Envs derived from short-term virus cultures in human lymphocytes or Env sequences derived from plasma vRNA/cDNA are a first approximation to T/F Envs, but they may differ in important but unrecognized features. Envs derived from extensively passaged virus cultures are less likely to reflect the biologic and antigenic properties of T/F viruses. In this context, 7 of the 10 new SHIVs described in the current study, and 12 of 16 SHIVs that we have reported overall (Table 1), were constructed using T/F Envs. The remainder was constructed using primary Envs.

A recent study by Keele and colleagues (29) aimed to create new subtype C T/F SHIVs using 20 South African subtype C T/F Envs and either of two strategies to enhance replication in primary RM CD4^+^ T cells. One of these strategies was the same EnvΔ375 design employed here and the other was an EnvΔ281 approach reported elsewhere (28). Because the O’Brien et al. study (29) pooled SHIVs for competitive replication analyses in RMs, a precise determination of the proportion of wild-type HIV-1 Envs that could support SHIV replication in monkeys could not be made. However, in the instances where EnvΔ375 substitutions were made and the resulting SHIVs were tested individually, EnvΔ375 substitutions were successful in conferring replication competence to SHIVs in rhesus cells. The addition of EnvΔ281 was neither additive nor synergistic. In our studies described here (Table 1) and elsewhere (35, 38), we created a total of 16 EnvΔ375 SHIVs, and each one replicated efficiently in RM CD4^+^ T cells *in vitro* and in RMs *in vivo*. Altogether, the results suggest that EnvΔ375 substitution is an effective means for creating SHIVs that have a high likelihood of replicating efficiently in RMs. The second-generation design strategy illustrated in Fig. 1B and Fig. 2 can facilitate this process by substantially reducing the time and effort required to construct new SHIVs and by improving their replication fitness.

A useful outbred primate model for HIV-1 infection of humans should be rational in design, amenable to iterative changes in the challenge viruses, and consistent in reproducing relevant features of disease. Previously, SHIV infections of RMs did not always meet these requirements since SHIVs replicated variably in RMs and often required *in vitro* or *in vivo* adaptations to achieve consistent infection or replication. Oftentimes, these changes were not fully understood mechanistically, nor were their immunobiological effects fully appreciated. Moreover, *in vitro* measures of virus content, infectivity, and replication in cell culture did not always predict *in vivo* outcomes, lending a measure of uncertainty to SHIV design and analysis. The EnvΔ375 strategy alleviates much of this uncertainty and unpredictability as demonstrated by the following results: (i) Env375 substitutions alone were sufficient to enhance Env affinity to rhesus CD4, reduce the energetic threshold for downstream Env transitions following CD4 binding, and convey efficient infectivity to the virus in primary rhesus CD4^+^ T cells *in vitro* and *in vivo*; (ii) the Env375 substitution strategy has worked consistently in that every attempt that we (Table 1), Keele (29), and Barouch (97) have made to engineer a T/F or primary HIV-1 Env SHIV by residue 375 substitution has succeeded in producing a chimeric virus that replicates efficiently in RMs; (iii) the ability of such EnvΔ375 SHIVs to replicate *in vivo* was, in each case, predicted by efficient replication in primary, activated rhesus CD4^+^ T cells *in vitro* and, as this is a different result from what has been reported for other SHIVs (1–3, 65), we suspect that our simple EnvΔ375 design scheme, our protocol for rhCD4+ T cell activation, and our method for infecting these cells in tissue culture are responsible for the differences; (iv) the antigenicity and tier 2 neutralization sensitivity of wild-type HIV-1 Envs was closely mirrored by EnvΔ375 mutants expressed from 293T cells or as infectious SHIVs from primary rhesus CD4 T cells; (v) the genetic diversity of each SHIV infection stock was very low when virus was expressed either from 293T cells or from primary rhesus CD4^+^ T cells; (vi) transmission efficiency of SHIVs across rhesus rectal, vaginal, penile, and oral mucosa, and intravenously, mirrored the transmission efficiency of HIV-1 in humans; (vii) acute and early SHIV replication dynamics in RMs measured by plasma vRNA replicated what has been seen in humans, including a 7 to 14 day eclipse period before vRNA is detectable in plasma, an exponential increase in plasma virus load to a peak approximately 14 to 28 days postinfection, establishment of setpoint viremia two or more months later, and immunopathogenesis leading to clinically defined AIDS in a subset of animals (69, 70, 73); (viii) SHIV-infected RMs consistently elicited autologous, strain-specific NAbs, and in some cases bNAbs, with kinetics similar to HIV-1-infected humans (35, 38); and (ix) molecular pathways of SHIV Env evolution in RMs closely mirrored evolution of homologous HIV-1 Envs in humans, including precise molecular patterns of Env-Ab coevolution leading to Nab escape and, in some animals, the development of bNAbs (38). The latter results speak to the native-like structure of SHIV Envs and to homologies and orthologies in human and rhesus immunoglobulin gene repertoires (38, 85). Altogether, the findings highlight the reproducibility and relevance of the SHIV EnvΔ375-infected RM as a model system for HIV-1 infection in humans. There are, however, limitations to the SHIV EnvΔ375-infected RM as a model for cure studies, since persistent virus replication is variable between different SHIVs and even with the same SHIV in different animals (Fig. 4) (35, 38). SHIVs D.191859, BG505.N332, and CH505 have generally shown the most consistent replication across multiple studies in monkeys that were not treated with anti-CD8 MAb; replication of these and other SHIVs was generally enhanced by about 10-fold by the administration of rhesus anti-CD8 MAb at the time of SHIV inoculation (Fig. 4). On the other hand, a mechanistic understanding of the basis of spontaneous control of SHIV replication in some monkeys but not others could conceivably inform studies of functional HIV-1 cure in humans.

Efficient mucosal transmission, leading to productive clinical infection with consistent patterns of plasma viremia, is a critical feature of SHIVs intended for use as challenge strains to test for vaccine efficacy and for mechanistic studies of virus transmission. We tested SHIVs BG505.N332, CH505, D.191859, and T250-4 for mucosal transmission and determined the titer of each challenge stock for 50% animal infectious doses (AID_50_). For these studies, virus challenge stocks were grown in primary rhesus CD4^+^ T cells. Challenge stocks were first subjected to thorough analytical measurements of virus concentration, infectivity, genotypic complexity, and phenotype with respect to coreceptor usage and antigenicity (Table 2, Fig. 7, Fig. 9). Because of the wide scientific interest of BG505.N332 SOSIP as a vaccine candidate, we conducted a low-dose atraumatic intrarectal (IR) titration study of SHIV.BG505.N332 (Fig. 10B) where we estimated the IR AID_50_ of this stock to be approximately 1:120 (1 ml). Burton and colleagues (7) corroborated this estimate by showing that 6 of 6 naive RMs inoculated intrarectally with a 1:20 (1 ml) dose of this same challenge stock, and 9 of 12 naive RMs inoculated intrarectally with a 1:75 (1 ml) dose, became productively infected after a single challenge (7). Importantly, these results demonstrated reproducibility in clinical infectivity titers of the identical challenge stock used at different primate centers and in animals obtained from different breeding colonies. Replication dynamics of SHIV.BG505.N332 following the low-dose intrarectal inoculations were quite similar in the two studies (Fig. 10B and reference 7): a meta-analysis of the results revealed peak viremia geometric mean titers of 2.7 × 10^6^ vRNA/ml at day 14 postchallenge and plasma viral load setpoint geometric mean titers of 9.2 × 10^3^ vRNA/ml by week 12, with 23 of 24 animals remaining viremic. Pulendran and colleagues used this same SHIV.BG505.N332 challenge stock for low-dose intravaginal (IVAG) challenges in a preclinical protection study in RMs (9). In a control arm of 15 sham-vaccinated RMs, they found the AID_50_ to be approximately 1:3 (1 ml). Peak plasma viremia (GMT = 1.7 × 10^6^ vRNA/ml) was again at 14 days postinfection and plasma viral load setpoint was reached by week 10, with 14 of 15 animals remaining viremic (GMT = 1.7 × 10^3^ vRNA/ml). The 40-fold difference in AID_50_ between IR and IVAG challenge routes is consistent with previous findings with SHIV and SIVs (3, 98, 99) and is similar to estimates of relative infectivity in humans exposed to receptive anal intercourse versus receptive vaginal intercourse (78). We also titrated SHIV.CH505 challenge stocks for AID_50_ in RMs following intrarectal or intravaginal inoculation. In independent studies with a total of 21 RMs, Klatt (reference 100 and unpublished data) and Haynes estimated the AID_50_ following IR challenge of naive RMs to be approximately 1:80 (1 ml), while Felber and colleagues (8) found the AID_50_ of this stock following IVAG challenge to be approximately 1:2 (1 ml) (Table 2). These findings again demonstrate reproducibility in AID_50_ titers in different primate centers and in monkeys from different breeding colonies, as well as a 30- to 40-fold difference in infectivity between IR versus IVAG challenge routes. Previously, we estimated the AID_50_ for SHIV.D.191879 for IVAG inoculation to be approximately 1:3 (1 ml) (101). Here, we could not estimate an AID_50_ for penile transmission by the SHIV.D.191879 challenge stock since 2 of 2 animals became infected after a single inoculation (Fig. 10A), but the findings suggest that the AID_50_ titers of this stock for penile transmission are likely to be sufficient for it to be used as a challenge stock in preclinical prevention trials once formal titering is completed. Finally, in an ongoing study, Sok, Rakasz, and colleagues have estimated the AID_50_ of SHIV.T250-4 to be approximately 1:160 (1 ml inoculum) following atraumatic rectal inoculation (unpublished data). Thus, in multiple studies of mucosal infection by BG505.N332, CH505, D.191859, and T250-4, AID_50_ titers and postinfection plasma viral load kinetics were consistent between SHIVs and between studies conducted at different primate facilities and mirrored analytical assessments of the different challenge stocks *in vitro* (Table 2). These findings suggest that precise measurements of virion content and infectivity of different challenge stocks correlate well with AID_50_ titers following mucosal challenge, which is important because it can facilitate AID_50_ titrations of new challenge stocks going forward.

Altogether, the findings of this study suggest that the SHIVs listed in Table 1 can be broadly useful as challenge stocks for preclinical studies of vaccine-elicited or passively acquired antibody protection, for assessing novel cure interventions, and for mechanistic studies of virus transmission and pathogenesis. We have contributed the rhCD4 T cell-grown SHIV challenge stocks and the 16 SHIV plasmid DNA stocks to the NIH NIAID HIV Reagent Repository and to the Penn Center for AIDS Research Virology Core Laboratory, which provide investigators with derivative reagents (e.g., barcoded SHIVs for lineage tracing, sequence-verified viral DNA maxipreps, minimally adapted T/F SHIV variants with enhanced *in vivo* replication dynamics, and titered 293T-derived infectious virus stocks) to meet research needs. One important research application that we anticipate in the future is in comparative efficacy testing of different vaccines against common heterologous tier 2 primary virus challenge stocks, and the same vaccine against a common heterologous virus administered by different mucosal inoculation routes. Such studies promise to inform HIV-1 immunogen design and testing as new vaccine candidates are developed.

## MATERIALS AND METHODS

### Ethical statement.

Indian rhesus macaques were housed and studied at Bioqual, Inc., Rockville, MD or at the Plum Borough animal facility at the University of Pittsburgh, Pittsburgh, PA, according to guidelines and standards of the Association for Assessment and Accreditation of Laboratory Animal Care and the Animal Welfare Act. Experiments were approved by the Bioqual, University of Pittsburgh, Duke University and University of Pennsylvania Institutional Animal Care and Use Committees. All RMs included in this study were socially housed (paired) indoors in stainless steel cages, had 12/12 light cycle, were fed twice daily, and water was provided *ad libitum*. A variety of recommended environmental enrichment strategies were employed. The animals were observed twice daily and any signs of disease or discomfort were reported to the veterinary staff for evaluation. For sample collections, animals were anesthetized with 10 mg/kg ketamine HCI (Park-Davis, Morris Plains, NJ, USA) or 0.7 mg/kg tiletamine (HCI) and zolazepan (Telazol, Fort Dodge Animal Health, Fort Dodge, IA) injected intramuscularly. At the end of the study, the animals were sacrificed by intravenous administration of barbiturates.

### Nonhuman primate care and procedures.

Animals were approximately equally divided male and female, aged 3 to 12 years and negative for Mamu-A*01, B*08, and B*17. Animals were screened to be negative for retroviruses, measles, Ebola virus, and T. cruzi. Animals were sedated for blood draws, anti-CD8 MAb infusions, and SHIV inoculations. For estimations of AID_50_ of SHIV challenge stocks, animals were inoculated atraumatically by penile, rectal, or vaginal routes. Penile inoculations were performed in anesthetized animals in a recumbent supine position. The foreskin was retracted vertically and laterally and separated from the glans exposing the preputial mucosa and coronal sulcus. An aliquot of 0.25 ml of undiluted SHIV challenge stock was slowly and carefully inoculated into this potential space using a 1-ml syringe and the vertical-lateral foreskin retraction maintained for 20 min. Intrarectal or intravaginal inoculations were performed by inserting a flexible lubricated pediatric feeding tube atraumatically 3 to 5 cm into the rectum or vagina of animals lying in a Trendelenburg position followed by the administration of diluted virus stock in a total volume of 1 ml by slow push. Animals were maintained in the Trendelenburg position for 30 min before being repositioned and returned to their cages to recover from anesthesia. Intravenous SHIV inoculations were performed by placing an intravenous (i.v.) catheter into the femoral vein of anesthetized animals in supine position, administering small volumes of sterile normal saline and confirming venous access by retrograde blood return. SHIVs bearing any of 10 different HIV-1 Envs, each with as many as six Env375 allelic variants in a total volume of 1 ml Dulbecco’s modified Eagle medium (DMEM), were administered by slow push. The different Env375 allelic variants were equilibrated based on p27Ag concentration, since the variable we were testing was relative infectivity as reflected in SHIV replication efficiency and plasma vRNA load *in vivo*. Thus, a typical inoculum consisted of a total of 300 ng SHIV p27Ag, composed of 50 ng p27Ag of each of six SHIV Env375 variants. Following virus inoculation, the i.v. line was flushed with normal saline. A subset of animals then received an intravenous infusion of 25 mg/kg of anti-CD8alpha MAb (MT807R1; NIH Nonhuman Primate Reagent Resource, NHPRR [https://www.nhpreagents.org/]) or anti-CD8beta MAb (CD8beta255R1; NHPRR) MAb over a period of 3 to 5 min. Another subset of animals received anti-CD8 MAb at 18 to 48 weeks postinfection. RMs were phlebotomized weekly, then monthly, and then every other month to collect and cryopreserve blood samples.

### Processing and storage of rhesus and human blood specimens.

Blood samples from rhesus macaques were collected in sterile 10-ml vacutainers containing ACD-A anticoagulant. Up to 40 ml of ACD-A anticoagulated blood from each RM was combined in a sterile 50-ml polypropylene conical tube, centrifuged at 2,100 rpm (1,000 × *g*) for 10 min at 20°C, and the plasma collected in a fresh 50 ml conical tube without disturbing the buffy coat WBC layer and large red cell pellet. The plasma was centrifuged again at 3,000 rpm (∼2,000 × *g*) for 15 min at 20°C in order to remove all platelets and cells. Plasma was collected and aliquoted into cryovials and stored at −80°C. The red blood cell (RBC)/WBC pellet was resuspended in an equal volume of Hanks balanced salt solution (HBSS) without Ca^++^ or Mg^++^ and containing 2 mM EDTA and then divided into four 50-ml conical tubes. Additional HBSS-EDTA (2 mM) buffer was added to bring the volume of the RBC/WBC mixture to 30 ml in each tube. The cell suspension was then carefully underlayered with 14 ml 96% Ficoll-Paque and centrifuged at 1,800 rpm (725 × *g*) for 20 min at 20°C in a swinging bucket tabletop centrifuge with slow acceleration and braking so as not to disrupt the Ficoll-cell interface. Mononuclear cells at the Ficoll interface were collected and transferred to a new 50-ml centrifuge tube containing HBSS-EDTA (w/o Ca^++^ or Mg^++^) and centrifuged at 1,000 rpm (∼200 × *g*) for 15 min at 20°C. This pellets peripheral blood mononuclear cells (PBMCs) and leaves most of the platelets in the supernatant. The supernatant was removed and the cell pellet was resuspended in 40 ml HBSS (with Mg^++^/Ca^++^ and without EDTA) + 1% fetal bovine serum (FBS). To remove additional contaminating platelets, the cell suspension was centrifuged again at 1,000 rpm (∼200 × *g*) for 15 min at 20°C and the supernatant discarded. The cell pellet was tap-resuspended in the residual 0.1 to 0.3 ml of medium and then brought to a volume of 10 ml HBSS (with Mg^++^/Ca^++^) plus 1% FBS. Cells were counted and viability assessed by trypan blue exclusion. Cells were centrifuged again at 1,200 rpm (300 × *g*) for 10 min at 20°C, the supernatant discarded, and the cells resuspended at a concentration of 5 to 10 × 10^6^ cells/ml in CryoStor cell cryopreservation medium (Sigma number C2999), aliquoted into 1-ml cryovials (CryoClear cryovials; Globe Scientific Inc., number 3010), placed in a Corning CoolCell LX cell freezing container, stored overnight at −80°C, and then transferred to vapor phase liquid N_2_ for long-term storage. Alternatively, freshly isolated rhesus PBMCs were processed immediately for CD4^+^ T cell purification and activation. Human PBMCs from deidentified normal blood samples were isolated by similar procedures from leukopaks obtained from the University of Pennsylvania Comprehensive Cancer Center Human Immunology Core Laboratory and either cryopreserved or used immediately for CD4^+^ T cell purification and activation.

### SHIV constructions.

SHIVs were constructed in one of two chimeric SIV/HIV proviral backbone plasmids. The original backbone (Fig. 1A) was first described by Li et al. (35) and was used in that study to generate SHIV.A.BG505, SHIV.B.YU2, SHIV.C.CH505, SHIV.C.CH848, and SHIV.D.191859. This backbone was subsequently employed by Roark to generate SHIV.C.CAP256SU (38) and by other investigators to generate still other SHIVs, all based on this EnvΔ375 design strategy (29, 97). We designated the first-generation plasmids as pCRXTOPO.SHIV.v1.backbone1 and pCRXTOPO.SHIV.v1.backbone2. Version 1 backbones 1 and 2 allow for the cloning of *vpu-env* (*env* nucleotides 1 to 2153, HXB2 numbering) or *env*-only (*env* nucleotides 1 to 2153, HXB2 numbering) amplicons, respectively. This first-generation plasmid required cumbersome sequential PCR amplifications and ligations in order to generate a complete replication-competent chimeric SHIV provirus. In addition, the first-generation design scheme generated an SIV/HIV-1 *tat1* redundancy and an HIV-1 *env* gp41 redundancy, both of which were spontaneously deleted when SHIVs replicated persistently *in vivo* (e.g., see Fig. 2A and references 35 and 38). We thus engineered second-generation SHIV cloning vectors designated pCRXTOPO.SHIV.v2.backbone1 and pCRXTOPO.SHIV.v2.backbone2, which allow for cloning of the identical *vpu-env* and *env*-only amplicons, respectively. In the first-generation SHIV backbone, unique restriction enzyme recognition sites for BstBI and XhoI are present in the middle of SIV *vpx* and after the 3′ LTR in the vector sequence, respectively. We synthesized two fragments that contain these two enzyme sites and the genes in between. We eliminated the redundant *tat1* and *env* gp41 sequences and replaced the *vpu-env* and *env* genes with a linker fragment that carries two BsmBI restriction enzyme sites (Fig. 1). The BsmBI site appended at the N terminus of the linker recognizes the reverse cDNA strand and creates a 3′ overhang; the one added at the C terminus recognizes the positive-strand DNA and creates a 5′ overhang. This design results in two different sticky ends, which allows unidirectional cloning of the insert into the backbone. BsmBI is a type IIS restriction enzyme that cleaves outside its recognition site and thus the enzyme recognition sequence does not remain after ligating the insert into the backbone (Fig. 1). The two synthesized fragments were then cloned into the original SHIV backbone separately using the BstBI and XhoI sites. The resulting two SHIV backbones (GenBank accession numbers MW476487 and MW476488) were then used for cloning *env* (nucleotides 1 to 2153, HXB2 numbering) or *vpu-env*, respectively. The *vpu*-*env* gp140 segments of HIV-1 CE1176, CH1012, T250-4, Q23.17, WITO, ZM233, 1086, B41, and 40100 were cloned into the first-generation SHIV backbone using methods described previously (Li, et al., 2016) (35). The *vpu*-*env* gp140 segments of HIV-1 CH0694 and CH505 were cloned into the second-generation SHIV backbone by appending the BsmBI recognition sequences to the 5′ and 3′ ends of the amplicon and performing a standard ligation (35). We then used the QuikChange II XL site-directed mutagenesis kit (Agilent Technologies) to create allelic variants (M, Y, F, W, or H) of the wild-type Env375-Ser or -Thr codons. Wild-type and mutant plasmids were transformed into MAX Efficiency Stbl2 competent cells (Invitrogen) for maxi-DNA preparations. Each 10-kb viral genome was sequenced in its entirety to authenticate its identity and genome integrity. Infectious SHIV stocks were generated in 293T cells as previously described.

### SHIV infection of primary rhesus and human CD4^+^ T cells.

Purified rhesus and human CD4^+^ T cells were isolated from PBMCs using magnetic MACS CD4 MicroBeads (Miltenyi Biotec), as previously described (35). They were activated by incubation with anti-biotin MACSiBead particles (Miltenyi Biotec) loaded with biotinylated anti-CD2, -CD28, and -CD3 MAbs, as previously described (35). The replication kinetics of each of the SHIVs and Env375 variants in primary, activated human and rhesus CD4^+^ T cells were determined again as previously described (35). Briefly, 293T supernatants containing 300 ng p27Ag of each variant were added to 2 × 10^6^ purified human or rhesus CD4 T cells in complete RPMI growth medium (RPMI 1640 with 15%) heat-inactivated fetal bovine serum (FBS, HyClone), 100 U/ml penicillin-streptomycin (Gibco), 30 U/ml interleukin 2 (IL-2) (aldesleukin, Prometheus Laboratories), and 30 μg/ml DEAE-dextran. For MOI estimation, 300 ng p27Ag is equal to ∼3 × 10^9^ virions, ∼3 × 10^5^ IU on TZM-bl cells, or ∼3 × 10^4^ IU on primary CD4^+^ T cells, so the estimated MOI of this titration was estimated to be between 0.01 and 0.05. The cell and virus mixtures were incubated for 2 h under constant rotation at 37°C to facilitate infection, washed three times with RPMI 1640, and resuspended in complete RPMI 1640 medium lacking DEAE-dextran. Cells were plated into 24-well plates at 2 million cells in 1 ml and cultured for 13 days, with sampling of 0.2 ml supernatant and medium replacement every 2 to 3 days. Supernatants were assayed for p27Ag concentration by enzyme-linked immunosorbent assay (ELISA) according to the manufacturer’s instructions (Zeptometrix).

### SHIV challenge stock generation in primary rhesus CD4^+^ T cells.

A total of 100 to 200 million primary, activated, rhesus CD4^+^ cells pooled from three naive RMs at a concentration of 10^7^ cells/ml in complete RPMI 1640 medium with 10% fetal calf serum (FCS) and DEAE-dextran (30 μg/ml) were inoculated with 293T cell-derived SHIVs at an MOI of 0.1 to 0.5 in TZM-bl cells and an estimated MOI of 0.01 to 0.05 in primary rhesus CD4^+^ T cells. For SHIV.CE1176, we infected primary rhesus cells with an equal mixture of Env375-His, -Phe, and -Trp alleles, and for SHIV.T250-4 we infected cells with an equal mixture of Env375-His, -Tyr, and -Trp alleles, because these alleles in these two Env backgrounds had shown differential replication in different animals (Fig. 3). The other SHIV challenge stocks were generated with viruses containing single rhesus-preferred Env375 alleles (Table 2). The total volume of the SHIV-cell mixture was typically 10 to 30 ml, depending of the infectivity titers of the 293T virus stock. The SHIV-cell mixture was transferred to a T75 flask, which was fixed to a rotating wheel or rocker so that leakage or spillage was not possible. This apparatus was then placed in a 37°C in a 5% CO_2_ incubator for 2 h of continuous mixing. The contents of the T75 flask were then transferred to a sterile 50-ml polypropylene tube and centrifuged at room temperature at 1,200 rpm (∼300 × *g*) for 10 min. The supernatant was decanted, the cells gently tap resuspended in the residual medium (<0.5 ml), and then resuspended in 50 ml complete RPMI medium with 10% FCS and the wash step repeated twice. The washing steps are important to remove DEAE-dextran, which can be toxic to cells in culture, and to remove unbound virus. Cells were then resuspended at a concentration of 1 to 2 × 10^6^ cells/ml in complete RPMI 1640 medium with 10% FCS, Il-2, and antibiotics in T100 flasks and incubated at 37°C in a 5% CO_2_ incubator. On days ∼7 and ∼14 post-SHIV inoculation, additional fresh medium and approximately 100 to 200 million fresh, uninfected, activated rhesus CD4^+^ T cells from three different naive RMs were added to the cultures, which were transferred into T250 flasks to accommodate larger volumes. This expansion of the cultures markedly increased cell numbers and supernatant volumes while maintaining cell concentrations between 2 and 4 million per milliliter. The culture supernatant was sampled on approximately days 1, 4, 7, 10, 14, 17, and 20 for p27Ag concentration, with assays performed weekly. Typically, p27Ag concentrations were <50 ng/ml on day 7 but rose rapidly to >200 ng/ml by day 10. On day ∼10 post-SHIV inoculation, the total volume of culture supernatant was collected, centrifuged twice at 2,500 rpm (1,000 × *g*) for 15 min to remove any residual cells or cell debris, and then frozen in bulk at −80°C. The supernatant was replaced with a greater volume of fresh medium as additional uninfected activated rhesus CD4^+^ T cells were added and as cells divided, again keeping cell concentrations at 2 to 4 million per milliliter. Between days 10 and 21, p27Ag production was maximal and concentrations in the supernatant rose rapidly to >200 ng/ml every 3 to 4 days after each complete collection of culture supernatant. By this means, we could collect as much as 2.5 liters of culture medium containing each SHIV over a 3-week culture period. Importantly, because complete supernatant collections and fresh media replacements were performed every 3 to 4 days beginning on day ∼10 post-SHIV inoculation, most of the virus that was collected and frozen was <4 days old and underwent only one freeze-thaw cycle prior to final vialing. Once all supernatant collections had been made over the 18- to 21-day culture period, they were thawed at room temperature at the same time, combined in a sterile 3-liter flask to ensure complete mixing, and aliquoted into as many as 2,500 cryovials, generally at 1 ml per vial. The vials were then transferred to a −80°C freezer overnight and then to vapor phase liquid nitrogen for long-term storage. By this means, we could ensure that all vials were essentially identical in their contents.

### Virus entry and neutralizing antibody assays.

Assays for virus entry and neutralizing antibodies were performed using TZM-bl indicator cells, as previously described (35, 91). The NAb assay is essentially identical to that employed by Montefiori, Seaman, and colleagues (102) (https://www.hiv.lanl.gov/content/nab-reference-strains/html/home.htm), the only difference being that we plate virus and test plasma or MAbs or purified polyclonal IgG onto adherent TZM-bl cells and hold the concentration of human and rhesus plasma/serum constant across all wells at 10%. In addition to this 10% final concentration of plasma/serum, the culture medium consists of Dulbecco’s modified Eagle’s medium (DMEM) with 40 μg/ml of DEAE-dextran and pen-strep antibiotics. Infections were performed in duplicate. Uninfected cells were used to correct for background luciferase activity. The infectivity of each virus without antibodies was set at 100%. The 50% inhibitory concentration (IC_50_) is the antibody concentration that reduces by 50% the relative light units (RLU) compared with the no-Ab control wells after correction for background. Nonlinear regression curves were determined and IC_50_ values calculated by using variable slope (four parameters) function in Prism software (v8.0). In the virus entry assay used to determine infectivity titers of 293T cell-derived viruses (Table 2), the culture medium consisted of DMEM with 10% FBS, 40 μg/ml DEAE-dextran, and pen-strep antibiotics and cell entry was quantified by beta-galactosidase expression after 48 h, as described (35).

### Coreceptor use analysis.

TZM-bl cells were seeded in 96-well plates at a density of 15,000 cells per well and cultured overnight at 37°C with humidified air and 5% CO_2_. Cells were incubated with selective entry inhibitors for 1 h, followed by inoculation of 2,000 TZM-bl IU of virus per well. Coreceptor inhibitors included 10 μM Maraviroc (CCR5), 1.2 μM AMD3100 (CXCR4), a mixture of inhibitors, or medium-only control. Viral Envs YU2 (CCR5-tropic) and SG3 (CXCR4-tropic) were included as controls. The infectivity of the medium-only control wells was set at 100%. The infectivity of the experimental wells was quantified by percentage of infection compared with the medium-only control wells after correction for background.

### Plasma viral RNA quantification.

Plasma viral load measurements were performed by the AIDS and Cancer Virus Program, Leidos Biomedical Research Inc., Frederick National Laboratory, and by the NIH/NIAID-sponsored Nonhuman Primate Virology Core Laboratory at the Duke Human Vaccine Institute, as previously described (35, 38). Over the course of this study, the sensitivity limits for accurate vRNA quantification using 0.5 ml of NHP plasma improved from 250 RNA cp/ml to <100 RNA cp/ml. We chose a conservative threshold of 100 RNA cp/ml for a limit of detection and 250 RNA cp/ml for the limit of quantification.

### Viral RNA sequencing.

Single genome sequencing of SHIV 3′ half genomes was performed as previously described (35, 73). Geneious software was used for alignments and sequence analysis. The sequences were visualized using the LANL Highlighter plot tools (https://www.hiv.lanl.gov/content/sequence/HIGHLIGHT/highlighter_top.html). To analyze the prevalence of 375 variants, next-generation sequencing was performed using the Illumina MiSeq system as described (35, 38). For each animal, 20,000 to 200,000 vRNA copies were used for reverse transcription and bulk reverse transcriptase PCR (RT-PCR). Raw reads from each bulk PCR were analyzed and the frequency of S, T, M, Y, H, W, and F codons at position 375 was determined by using Geneious software.

### Statistical analyses.

Statistical tests were calculated using GraphPad Prism 8 software. The Mann-Whitney test was used to determine whether the peak and setpoint viral loads of anti-CD8-treated animals were significantly different from untreated animals. We chose a nonparametric rank-based test because both peak and setpoint viral loads of the untreated group failed the D’Agostino & Pearson normality test (*P* values < 0.05). The geometric means were calculated using the Column statistics function of GraphPad Prism 8. The mean and maximum diversities were calculated using the Poisson-Fitter v2 program (https://www.hiv.lanl.gov).

### Data availability.

Sequences determined in the present study are available in GenBank under accession numbers KU958487, MW410732 to MW410742, MW476487 and MW476488, MW484951 to MW484987, and MW507842 to MW508333.

## References

[1] Hatziioannou T, Evans DT. 2012. Animal models for HIV/AIDS research. Nat Rev Microbiol 10:852–867. 10.1038/nrmicro2911. 23154262 PMC4334372

[2] Sharma A, Boyd DF, Overbaugh J. 2015. Development of SHIVs with circulating, transmitted HIV-1 variants. J Med Primatol 44:296–300. 10.1111/jmp.12179. 26101933

[3] Del Prete GQ, Lifson JD, Keele BF. 2016. Nonhuman primate models for the evaluation of HIV-1 preventive vaccine strategies: model parameter considerations and consequences. Curr Opin HIV AIDS 11:546–554. 10.1097/COH.0000000000000311. 27559710 PMC5100008

[4] Hessell AJ, Poignard P, Hunter M, Hangartner L, Tehrani DM, Bleeker WK, Parren PW, Marx PA, Burton DR. 2009. Effective, low-titer antibody protection against low-dose repeated mucosal SHIV challenge in macaques. Nat Med 15:951–954. 10.1038/nm.1974. 19525965 PMC4334439

[5] Hessell AJ, Malherbe DC, Haigwood NL. 2018. Passive and active antibody studies in primates to inform HIV vaccines. Expert Rev Vaccines 17:127–144. 10.1080/14760584.2018.1425619. 29307225 PMC6587971

[6] Pauthner M, Havenar-Daughton C, Sok D, Nkolola JP, Bastidas R, Boopathy AV, Carnathan DG, Chandrashekar A, Cirelli KM, Cottrell CA, Eroshkin AM, Guenaga J, Kaushik K, Kulp DW, Liu J, McCoy LE, Oom AL, Ozorowski G, Post KW, Sharma SK, Steichen JM, de Taeye SW, Tokatlian T, Torrents d. l P A, Butera ST, LaBranche CC, Montefiori DC, Silvestri G, Wilson IA, Irvine DJ, Sanders RW, Schief WR, Ward AB, Wyatt RT, Barouch DH, Crotty S, Burton DR. 2017. Elicitation of robust tier 2 neutralizing antibody responses in non-human primates by HIV envelope trimer immunization using optimized approaches. Immunity 46:1073–1088.E8. 10.1016/j.immuni.2017.05.007. 28636956 PMC5483234

[7] Pauthner MG, Nkolola JP, Havenar-Daughton C, Murrell B, Reiss SM, Bastidas R, Prevost J, Nedellec R, von Bredow B, Abbink P, Cottrell CA, Kulp DW, Tokatlian T, Nogal B, Bianchi M, Li H, Lee JH, Butera ST, Evans DT, Hangartner L, Finzi A, Wilson IA, Wyatt RT, Irvine DJ, Schief WR, Ward AB, Sanders RW, Crotty S, Shaw GM, Barouch DH, Burton DR. 2019. Vaccine-induced protection from homologous tier 2 SHIV challenge in non-human primates depends on serum-neutralizing antibody titers. Immunity 50:241–252.E6. 10.1016/j.immuni.2018.11.011. 30552025 PMC6335502

[8] Felber BK, Lu Z, Hu X, Valentin A, Rosati M, Remmel CAL, Weiner JA, Carpenter MC, Faircloth K, Stanfield-Oakley S, Williams WB, Shen X, Tomaras GD, LaBranche CC, Montefiori D, Trinh HV, Rao M, Alam MS, Vandergrift NA, Saunders KO, Wang Y, Rountree W, Das J, Alter G, Reed SG, Aye PP, Schiro F, Pahar B, Dufour JP, Veazey RS, Marx PA, Venzon DJ, Shaw GM, Ferrari G, Ackerman ME, Haynes BF, Pavlakis GN. 2020. Co-immunization of DNA and protein in the same anatomical sites Induces superior protective immune responses against SHIV challenge. Cell Rep 31:107624. 10.1016/j.celrep.2020.107624. 32402293 PMC7329227

[9] Arunachalam PS, Charles TP, Joag V, Bollimpelli VS, Scott MKD, Wimmers F, Burton SL, Labranche CC, Petitdemange C, Gangadhara S, Styles TM, Quarnstrom CF, Walter KA, Ketas TJ, Legere T, Jagadeesh Reddy PB, Kasturi SP, Tsai A, Yeung BZ, Gupta S, Tomai M, Vasilakos J, Shaw GM, Kang CY, Moore JP, Subramaniam S, Khatri P, Montefiori D, Kozlowski PA, Derdeyn CA, Hunter E, Masopust D, Amara RR, Pulendran B. 2020. T cell-inducing vaccine durably prevents mucosal SHIV infection even with lower neutralizing antibody titers. Nat Med 26:932–940. 10.1038/s41591-020-0858-8. 32393800 PMC7303014

[10] Pegu A, Borate B, Huang Y, Pauthner MG, Hessell AJ, Julg B, Doria-Rose NA, Schmidt SD, Carpp LN, Cully MD, Chen X, Shaw GM, Barouch DH, Haigwood NL, Corey L, Burton DR, Roederer M, Gilbert PB, Mascola JR, Huang Y. 2019. A meta-analysis of passive immunization studies shows that serum-neutralizing antibody titer associates with protection against SHIV challenge. Cell Host Microbe 26:336–346.E3. 10.1016/j.chom.2019.08.014. 31513771 PMC6755677

[11] Corey L, Gilbert PB, Juraska M, Montefiori DC, Morris L, Karuna ST, Edupuganti S, Mgodi NM, deCamp AC, Rudnicki E, Huang Y, Gonzales P, Cabello R, Orrell C, Lama JR, Laher F, Lazarus EM, Sanchez J, Frank I, Hinojosa J, Sobieszczyk ME, Marshall KE, Mukwekwerere PG, Makhema J, Baden L, Mullins JI, Williamson C, Hural J, McElrath J, Bentley C, Takuva S, Lorenzo MMG, Burns DN, Espy N, Randhawa AK, Kochar N, Donnell DJ, Sista N, Andrew P, Kublin JG, Gray G, Ledgerwood JE, Mascola JR, Cohen MS. 2021. Antibody mediated prevention of HIV-1 acquisition in high-risk populations by the broadly neutralizing antibody VRC01. N Engl J Med 384:1003–1014. 10.1056/NEJMoa2031738. 33730454 PMC8189692

[12] Barouch DH, Whitney JB, Moldt B, Klein F, Oliveira TY, Liu J, Stephenson KE, Chang HW, Shekhar K, Gupta S, Nkolola JP, Seaman MS, Smith KM, Borducchi EN, Cabral C, Smith JY, Blackmore S, Sanisetty S, Perry JR, Beck M, Lewis MG, Rinaldi W, Chakraborty AK, Poignard P, Nussenzweig MC, Burton DR. 2013. Therapeutic efficacy of potent neutralizing HIV-1-specific monoclonal antibodies in SHIV-infected rhesus monkeys. Nature 503:224–228. 10.1038/nature12744. 24172905 PMC4017780

[13] Shingai M, Nishimura Y, Klein F, Mouquet H, Donau OK, Plishka R, Buckler-White A, Seaman M, Piatak M, Jr., Lifson JD, Dimitrov DS, Nussenzweig MC, Martin MA. 2013. Antibody-mediated immunotherapy of macaques chronically infected with SHIV suppresses viraemia. Nature 503:277–280. 10.1038/nature12746. 24172896 PMC4133787

[14] Borducchi EN, Liu J, Nkolola JP, Cadena AM, Yu WH, Fischinger S, Broge T, Abbink P, Mercado NB, Chandrashekar A, Jetton D, Peter L, McMahan K, Moseley ET, Bekerman E, Hesselgesser J, Li W, Lewis MG, Alter G, Geleziunas R, Barouch DH. 2018. Antibody and TLR7 agonist delay viral rebound in SHIV-infected monkeys. Nature 563:360–364. 10.1038/s41586-018-0600-6. 30283138 PMC6237629

[15] Bender AM, Simonetti FR, Kumar MR, Fray EJ, Bruner KM, Timmons AE, Tai KY, Jenike KM, Antar AAR, Liu PT, Ho YC, Raugi DN, Seydi M, Gottlieb GS, Okoye AA, Del Prete GQ, Picker LJ, Mankowski JL, Lifson JD, Siliciano JD, Laird GM, Barouch DH, Clements JE, Siliciano RF. 2019. The landscape of persistent viral genomes in ART-treated SIV, SHIV, and HIV-2 infections. Cell Host Microbe 26:73–84.E4. 10.1016/j.chom.2019.06.005. 31295427 PMC6724192

[16] Dashti A, Waller C, Mavigner M, Schoof N, Bar KJ, Shaw GM, Vanderford TH, Liang S, Lifson JD, Dunham RM, Ferrari G, Tuyishime M, Lam CK, Nordstrom JL, Margolis DM, Silvestri G, Chahroudi A. 2020. SMAC mimetic plus triple-combination bispecific HIVxCD3 retargeting molecules in SHIV.C.CH505-infected, antiretroviral therapy-suppressed rhesus macaques. J Virol 94:e00793-20. 10.1128/JVI.00793-20. 32817214 PMC7565632

[17] Obregon-Perko V, Bricker K, Mensah G, Uddin F, Kumar M, Fray E, Siliciano RF, Schoof N, Horner A, Mavigner M, Liang S, Vanderford T, Sass J, Chan C, Berendam SJ, Bar K, Shaw GM, Silvestri G, Fouda G, Permar S, Chahroudi A. 2020. SHIV.C.CH505 persistence in ART-suppressed infant macaques is characterized by elevated SHIV RNA in the gut and high abundance of intact SHIV DNA in naive CD4+ T cells. J Virol 95:e01669-20. 10.1128/JVI.01669-20. 33087463 PMC7944446

[18] Harper J, Gordon S, Chan CN, Wang H, Lindemuth E, Galardi C, Falcinelli SD, Raines SLM, Read JL, Nguyen K, McGary CS, Nekorchuk M, Busman-Sahay K, Schawalder J, King C, Pino M, Micci L, Cervasi B, Jean S, Sanderson A, Johns B, Koblansky AA, Amrine-Madsen H, Lifson J, Margolis DM, Silvestri G, Bar KJ, Favre D, Estes JD, Paiardini M. 2020. CTLA-4 and PD-1 dual blockade induces SIV reactivation without control of rebound after antiretroviral therapy interruption. Nat Med 26:519–528. 10.1038/s41591-020-0782-y. 32284611 PMC7790171

[19] Saunders KO, Verkoczy LK, Jiang C, Zhang J, Parks R, Chen H, Housman M, Bouton-Verville H, Shen X, Trama AM, Scearce R, Sutherland L, Santra S, Newman A, Eaton A, Xu K, Georgiev IS, Joyce MG, Tomaras GD, Bonsignori M, Reed SG, Salazar A, Mascola JR, Moody MA, Cain DW, Centlivre M, Zurawski S, Zurawski G, Erickson HP, Kwong PD, Alam SM, Levy Y, Montefiori DC, Haynes BF. 2017. Vaccine induction of heterologous Tier 2 HIV-1 neutralizing antibodies in animal models. Cell Rep 21:3681–3690. 10.1016/j.celrep.2017.12.028. 29281818 PMC5777169

[20] Voss JE, Andrabi R, McCoy LE, de Val N, Fuller RP, Messmer T, Su CY, Sok D, Khan SN, Garces F, Pritchard LK, Wyatt RT, Ward AB, Crispin M, Wilson IA, Burton DR. 2017. Elicitation of neutralizing antibodies targeting the V2 apex of the HIV envelope trimer in a wild-type animal model. Cell Rep 21:222–235. 10.1016/j.celrep.2017.09.024. 28978475 PMC5640805

[21] Li J, Lord CI, Haseltine W, Letvin NL, Sodroski J. 1992. Infection of cynomolgus monkeys with a chimeric HIV-1/SIVmac virus that expresses the HIV-1 envelope glycoproteins. J Acquir Immune Defic Syndr (1988) 5:639–646. 1613662

[22] Karlsson GB, Halloran M, Li J, Park IW, Gomila R, Reimann KA, Axthelm MK, Iliff SA, Letvin NL, Sodroski J. 1997. Characterization of molecularly cloned simian-human immunodeficiency viruses causing rapid CD4+ lymphocyte depletion in rhesus monkeys. J Virol 71:4218–4225. 10.1128/JVI.71.6.4218-4225.1997. 9151808 PMC191636

[23] Harouse JM, Gettie A, Eshetu T, Tan RC, Bohm R, Blanchard J, Baskin G, Cheng-Mayer C. 2001. Mucosal transmission and induction of simian AIDS by CCR5-specific simian/human immunodeficiency virus SHIV(SF162P3). J Virol 75:1990–1995. 10.1128/JVI.75.4.1990-1995.2001. 11160699 PMC115146

[24] Song RJ, Chenine AL, Rasmussen RA, Ruprecht CR, Mirshahidi S, Grisson RD, Xu W, Whitney JB, Goins LM, Ong H, Li PL, Shai-Kobiler E, Wang T, McCann CM, Zhang H, Wood C, Kankasa C, Secor WE, McClure HM, Strobert E, Else JG, Ruprecht RM. 2006. Molecularly cloned SHIV-1157ipd3N4: a highly replication-competent, mucosally transmissible R5 simian-human immunodeficiency virus encoding HIV clade C Env. J Virol 80:8729–8738. 10.1128/JVI.00558-06. 16912320 PMC1563858

[25] Nishimura Y, Shingai M, Willey R, Sadjadpour R, Lee WR, Brown CR, Brenchley JM, Buckler-White A, Petros R, Eckhaus M, Hoffman V, Igarashi T, Martin MA. 2010. Generation of the pathogenic R5-tropic simian/human immunodeficiency virus SHIVAD8 by serial passaging in rhesus macaques. J Virol 84:4769–4781. 10.1128/JVI.02279-09. 20147396 PMC2863788

[26] Warren CJ, Meyerson NR, Dirasantha O, Feldman ER, Wilkerson GK, Sawyer SL. 2019. Selective use of primate CD4 receptors by HIV-1. PLoS Biol 17:e3000304. 10.1371/journal.pbio.3000304. 31181085 PMC6586362

[27] Humes D, Emery S, Laws E, Overbaugh J. 2012. A species-specific amino acid difference in the macaque CD4 receptor restricts replication by global circulating HIV-1 variants representing viruses from recent infection. J Virol 86:12472–12483. 10.1128/JVI.02176-12. 22973036 PMC3497638

[28] Del Prete GQ, Keele BF, Fode J, Thummar K, Swanstrom AE, Rodriguez A, Raymond A, Estes JD, LaBranche CC, Montefiori DC, KewalRamani VN, Lifson JD, Bieniasz PD, Hatziioannou T. 2017. A single gp120 residue can affect HIV-1 tropism in macaques. PLoS Pathog 13:e1006572. 10.1371/journal.ppat.1006572. 28945790 PMC5629034

[29] O'Brien SP, Swanstrom AE, Pegu A, Ko SY, Immonen TT, Del Prete GQ, Fennessey CM, Gorman J, Foulds KE, Schmidt SD, Doria-Rose N, Williamson C, Hatziioannou T, Bieniasz PD, Li H, Shaw GM, Mascola JR, Koup RA, Kwong PD, Lifson JD, Roederer M, Keele BF. 2019. Rational design and in vivo selection of SHIVs encoding transmitted/founder subtype C HIV-1 envelopes. PLoS Pathog 15:e1007632. 10.1371/journal.ppat.1007632. 30943274 PMC6447185

[30] Finzi A, Pacheco B, Xiang SH, Pancera M, Herschhorn A, Wang L, Zeng X, Desormeaux A, Kwong PD, Sodroski J. 2012. Lineage-specific differences between human and simian immunodeficiency virus regulation of gp120 trimer association and CD4 binding. J Virol 86:8974–8986. 10.1128/JVI.01076-12. 22696649 PMC3416166

[31] Xiang SH, Kwong PD, Gupta R, Rizzuto CD, Casper DJ, Wyatt R, Wang L, Hendrickson WA, Doyle ML, Sodroski J. 2002. Mutagenic stabilization and/or disruption of a CD4-bound state reveals distinct conformations of the human immunodeficiency virus type 1 gp120 envelope glycoprotein. J Virol 76:9888–9899. 10.1128/jvi.76.19.9888-9899.2002. 12208966 PMC136507

[32] Ding S, Medjahed H, Prevost J, Coutu M, Xiang SH, Finzi A. 2016. Lineage-specific differences between the gp120 inner domain layer 3 of human immunodeficiency virus and that of simian immunodeficiency virus. J Virol 90:10065–10073. 10.1128/JVI.01215-16. 27535053 PMC5105645

[33] Prevost J, Zoubchenok D, Richard J, Veillette M, Pacheco B, Coutu M, Brassard N, Parsons MS, Ruxrungtham K, Bunupuradah T, Tovanabutra S, Hwang KK, Moody MA, Haynes BF, Bonsignori M, Sodroski J, Kaufmann DE, Shaw GM, Chenine AL, Finzi A. 2017. Influence of the envelope gp120 phe 43 cavity on HIV-1 sensitivity to antibody-dependent cell-mediated cytotoxicity responses. J Virol 91:e02452-16. 10.1128/JVI.02452-16. 28100618 PMC5355605

[34] Prevost J, Tolbert WD, Medjahed H, Sherburn RT, Madani N, Zoubchenok D, Gendron-Lepage G, Gaffney AE, Grenier MC, Kirk S, Vergara N, Han C, Mann BT, Chenine AL, Ahmed A, Chaiken I, Kirchhoff F, Hahn BH, Haim H, Abrams CF, Smith AB, III, Sodroski J, Pazgier M, Finzi A. 2020. The HIV-1 Env gp120 inner domain shapes the phe43 cavity and the CD4 binding site. mBio 11:e00280-20. 10.1128/mBio.00280-20. 32457241 PMC7251204

[35] Li H, Wang S, Kong R, Ding W, Lee FH, Parker Z, Kim E, Learn GH, Hahn P, Policicchio B, Brocca-Cofano E, Deleage C, Hao X, Chuang GY, Gorman J, Gardner M, Lewis MG, Hatziioannou T, Santra S, Apetrei C, Pandrea I, Alam SM, Liao HX, Shen X, Tomaras GD, Farzan M, Chertova E, Keele BF, Estes JD, Lifson JD, Doms RW, Montefiori DC, Haynes BF, Sodroski JG, Kwong PD, Hahn BH, Shaw GM. 2016. Envelope residue 375 substitutions in simian-human immunodeficiency viruses enhance CD4 binding and replication in rhesus macaques. Proc Natl Acad Sci U S A 113:E3413–22. 10.1073/pnas.1606636113. 27247400 PMC4914158

[36] Desormeaux A, Coutu M, Medjahed H, Pacheco B, Herschhorn A, Gu C, Xiang SH, Mao Y, Sodroski J, Finzi A. 2013. The highly conserved layer-3 component of the HIV-1 gp120 inner domain is critical for CD4-required conformational transitions. J Virol 87:2549–2562. 10.1128/JVI.03104-12. 23255784 PMC3571356

[37] Finzi A, Xiang SH, Pacheco B, Wang L, Haight J, Kassa A, Danek B, Pancera M, Kwong PD, Sodroski J. 2010. Topological layers in the HIV-1 gp120 inner domain regulate gp41 interaction and CD4-triggered conformational transitions. Mol Cell 37:656–667. 10.1016/j.molcel.2010.02.012. 20227370 PMC2854584

[38] Roark RS, Li H, Williams WB, Chug H, Mason RD, Gorman J, Wang S, Lee FH, Rando J, Bonsignori M, Hwang KK, Saunders KO, Wiehe K, Moody MA, Hraber PT, Wagh K, Giorgi EE, Russell RM, Bibollet-Ruche F, Liu W, Connell J, Smith AG, DeVoto J, Murphy AI, Smith J, Ding W, Zhao C, Chohan N, Okumura M, Rosario C, Ding Y, Lindemuth E, Bauer AM, Bar KJ, Ambrozak D, Chao CW, Chuang GY, Geng H, Lin BC, Louder MK, Nguyen R, Zhang B, Lewis MG, Raymond D, Doria-Rose NA, Schramm CA, Douek DC, Roederer M, Kepler TB, Kelsoe G, . 2021. Recapitulation of HIV-1 Env-antibody coevolution in macaques leading to neutralization breadth. Science 371:eabd2638. 10.1126/science.abd2638. 33214287 PMC8040783

[39] Poss M, Overbaugh J. 1999. Variants from the diverse virus population identified at seroconversion of a clade A human immunodeficiency virus type 1-infected woman have distinct biological properties. J Virol 73:5255–5264. 10.1128/JVI.73.7.5255-5264.1999. 10364271 PMC112580

[40] Seaman MS, Janes H, Hawkins N, Grandpre LE, Devoy C, Giri A, Coffey RT, Harris L, Wood B, Daniels MG, Bhattacharya T, Lapedes A, Polonis VR, McCutchan FE, Gilbert PB, Self SG, Korber BT, Montefiori DC, Mascola JR. 2010. Tiered categorization of a diverse panel of HIV-1 Env pseudoviruses for assessment of neutralizing antibodies. J Virol 84:1439–1452. 10.1128/JVI.02108-09. 19939925 PMC2812321

[41] deCamp A, Hraber P, Bailer RT, Seaman MS, Ochsenbauer C, Kappes J, Gottardo R, Edlefsen P, Self S, Tang H, Greene K, Gao H, Daniell X, Sarzotti-Kelsoe M, Gorny MK, Zolla-Pazner S, LaBranche CC, Mascola JR, Korber BT, Montefiori DC. 2014. Global panel of HIV-1 Env reference strains for standardized assessments of vaccine-elicited neutralizing antibodies. J Virol 88:2489–2507. 10.1128/JVI.02853-13. 24352443 PMC3958090

[42] Hraber P, Seaman MS, Bailer RT, Mascola JR, Montefiori DC, Korber BT. 2014. Prevalence of broadly neutralizing antibody responses during chronic HIV-1 infection. AIDS 28:163–169. 10.1097/QAD.0000000000000106. 24361678 PMC4042313

[43] Abrahams MR, Anderson JA, Giorgi EE, Seoighe C, Mlisana K, Ping LH, Athreya GS, Treurnicht FK, Keele BF, Wood N, Salazar-Gonzalez JF, Bhattacharya T, Chu H, Hoffman I, Galvin S, Mapanje C, Kazembe P, Thebus R, Fiscus S, Hide W, Cohen MS, Karim SA, Haynes BF, Shaw GM, Hahn BH, Korber BT, Swanstrom R, Williamson C, CAPRISA Acute Infection Study Team, Center for HIV-AIDS Vaccine Immunology Consortium. 2009. Quantitating the multiplicity of infection with human immunodeficiency virus type 1 subtype C reveals a non-poisson distribution of transmitted variants. J Virol 83:3556–3567. 10.1128/JVI.02132-08. 19193811 PMC2663249

[44] Liao HX, Tsao CY, Alam SM, Muldoon M, Vandergrift N, Ma BJ, Lu X, Sutherland LL, Scearce RM, Bowman C, Parks R, Chen H, Blinn JH, Lapedes A, Watson S, Xia SM, Foulger A, Hahn BH, Shaw GM, Swanstrom R, Montefiori DC, Gao F, Haynes BF, Korber B. 2013. Antigenicity and immunogenicity of transmitted/founder, consensus, and chronic envelope glycoproteins of human immunodeficiency virus type 1. J Virol 87:4185–4201. 10.1128/JVI.02297-12. 23365441 PMC3624376

[45] Alexander J, Mendy J, Vang L, Avanzini JB, Garduno F, Manayani DJ, Ishioka G, Farness P, Ping LH, Swanstrom R, Parks R, Liao HX, Haynes BF, Montefiori DC, LaBranche C, Smith J, Gurwith M, Mayall T. 2013. Pre-clinical development of a recombinant, replication-competent adenovirus serotype 4 vector vaccine expressing HIV-1 envelope 1086 clade C. PLoS One 8:e82380. 10.1371/journal.pone.0082380. 24312658 PMC3849430

[46] Zambonelli C, Dey AK, Hilt S, Stephenson S, Go EP, Clark DF, Wininger M, Labranche C, Montefiori D, Liao HX, Swanstrom RI, Desaire H, Haynes BF, Carfi A, Barnett SW. 2016. Generation and characterization of a bivalent HIV-1 Subtype C gp120 protein boost for proof-of-concept HIV vaccine efficacy trials in southern Africa. PLoS One 11:e0157391. 10.1371/journal.pone.0157391. 27442017 PMC4956256

[47] Pugach P, Ozorowski G, Cupo A, Ringe R, Yasmeen A, de Val N, Derking R, Kim HJ, Korzun J, Golabek M, de Los Reyes K, Ketas TJ, Julien JP, Burton DR, Wilson IA, Sanders RW, Klasse PJ, Ward AB, Moore JP. 2015. A native-like SOSIP.664 trimer based on an HIV-1 subtype B env gene. J Virol 89:3380–3395. 10.1128/JVI.03473-14. 25589637 PMC4337520

[48] Kijak GH, Sanders-Buell E, Chenine AL, Eller MA, Goonetilleke N, Thomas R, Leviyang S, Harbolick EA, Bose M, Pham P, Oropeza C, Poltavee K, O'Sullivan AM, Billings E, Merbah M, Costanzo MC, Warren JA, Slike B, Li H, Peachman KK, Fischer W, Gao F, Cicala C, Arthos J, Eller LA, O'Connell RJ, Sinei S, Maganga L, Kibuuka H, Nitayaphan S, Rao M, Marovich MA, Krebs SJ, Rolland M, Korber BT, Shaw GM, Michael NL, Robb ML, Tovanabutra S, Kim JH. 2017. Rare HIV-1 transmitted/founder lineages identified by deep viral sequencing contribute to rapid shifts in dominant quasispecies during acute and early infection. PLoS Pathog 13:e1006510. 10.1371/journal.ppat.1006510. 28759651 PMC5552316

[49] Rolland M, Tovanabutra S, Dearlove B, Li Y, Owen CL, Lewitus E, Sanders-Buell E, Bose M, O'Sullivan A, Rossenkhan R, Labuschagne JPL, Edlefsen PT, Reeves DB, Kijak G, Miller S, Poltavee K, Lee J, Bonar L, Harbolick E, Ahani B, Pham P, Kibuuka H, Maganga L, Nitayaphan S, Sawe FK, Eller LA, Gramzinski R, Kim JH, Michael NL, Robb ML, RV217 Study Team. 2020. Molecular dating and viral load growth rates suggested that the eclipse phase lasted about a week in HIV-1 infected adults in East Africa and Thailand. PLoS Pathog 16:e1008179. 10.1371/journal.ppat.1008179. 32027734 PMC7004303

[50] Wagh K, Kreider EF, Li Y, Barbian HJ, Learn GH, Giorgi E, Hraber PT, Decker TG, Smith AG, Gondim MV, Gillis L, Wandzilak J, Chuang GY, Rawi R, Cai F, Pellegrino P, Williams I, Overbaugh J, Gao F, Kwong PD, Haynes BF, Shaw GM, Borrow P, Seaman MS, Hahn BH, Korber B. 2018. Completeness of HIV-1 envelope glycan shield at transmission determines neutralization breadth. Cell Rep 25:893–908.E7. 10.1016/j.celrep.2018.09.087. 30355496 PMC6426304

[51] Rademeyer C, Korber B, Seaman MS, Giorgi EE, Thebus R, Robles A, Sheward DJ, Wagh K, Garrity J, Carey BR, Gao H, Greene KM, Tang H, Bandawe GP, Marais JC, Diphoko TE, Hraber P, Tumba N, Moore PL, Gray GE, Kublin J, McElrath MJ, Vermeulen M, Middelkoop K, Bekker LG, Hoelscher M, Maboko L, Makhema J, Robb ML, Abdool Karim S, Abdool Karim Q, Kim JH, Hahn BH, Gao F, Swanstrom R, Morris L, Montefiori DC, Williamson C. 2016. Features of recently transmitted HIV-1 clade C viruses that impact antibody recognition: implications for active and passive immunization. PLoS Pathog 12:e1005742. 10.1371/journal.ppat.1005742. 27434311 PMC4951126

[52] Bonsignori M, Hwang KK, Chen X, Tsao CY, Morris L, Gray E, Marshall DJ, Crump JA, Kapiga SH, Sam NE, Sinangil F, Pancera M, Yongping Y, Zhang B, Zhu J, Kwong PD, O'Dell S, Mascola JR, Wu L, Nabel GJ, Phogat S, Seaman MS, Whitesides JF, Moody MA, Kelsoe G, Yang X, Sodroski J, Shaw GM, Montefiori DC, Kepler TB, Tomaras GD, Alam SM, Liao HX, Haynes BF. 2011. Analysis of a clonal lineage of HIV-1 envelope V2/V3 conformational epitope-specific broadly neutralizing antibodies and their inferred unmutated common ancestors. J Virol 85:9998–10009. 10.1128/JVI.05045-11. 21795340 PMC3196428

[53] Andrabi R, Voss JE, Liang CH, Briney B, McCoy LE, Wu CY, Wong CH, Poignard P, Burton DR. 2015. Identification of common features in prototype broadly neutralizing antibodies to HIV envelope V2 apex to facilitate vaccine design. Immunity 43:959–973. 10.1016/j.immuni.2015.10.014. 26588781 PMC4654981

[54] Gorman J, Soto C, Yang MM, Davenport TM, Guttman M, Bailer RT, Chambers M, Chuang GY, DeKosky BJ, Doria-Rose NA, Druz A, Ernandes MJ, Georgiev IS, Jarosinski MC, Joyce MG, Lemmin TM, Leung S, Louder MK, McDaniel JR, Narpala S, Pancera M, Stuckey J, Wu X, Yang Y, Zhang B, Zhou T, Program NCS, Mullikin JC, Baxa U, Georgiou G, McDermott AB, Bonsignori M, Haynes BF, Moore PL, Morris L, Lee KK, Shapiro L, Mascola JR, Kwong PD, NISC Comparative Sequencing Program. 2016. Structures of HIV-1 Env V1V2 with broadly neutralizing antibodies reveal commonalities that enable vaccine design. Nat Struct Mol Biol 23:81–90. 10.1038/nsmb.3144. 26689967 PMC4833398

[55] Wu X, Parast AB, Richardson BA, Nduati R, John-Stewart G, Mbori-Ngacha D, Rainwater SM, Overbaugh J. 2006. Neutralization escape variants of human immunodeficiency virus type 1 are transmitted from mother to infant. J Virol 80:835–844. 10.1128/JVI.80.2.835-844.2006. 16378985 PMC1346878

[56] Goo L, Chohan V, Nduati R, Overbaugh J. 2014. Early development of broadly neutralizing antibodies in HIV-1-infected infants. Nat Med 20:655–658. 10.1038/nm.3565. 24859529 PMC4060046

[57] Li Y, Kappes JC, Conway JA, Price RW, Shaw GM, Hahn BH. 1991. Molecular characterization of human immunodeficiency virus type 1 cloned directly from uncultured human brain tissue: identification of replication-competent and -defective viral genomes. J Virol 65:3973–3985. 10.1128/JVI.65.8.3973-3985.1991. 1830110 PMC248827

[58] Li Y, Hui H, Burgess CJ, Price RW, Sharp PM, Hahn BH, Shaw GM. 1992. Complete nucleotide sequence, genome organization, and biological properties of human immunodeficiency virus type 1 in vivo: evidence for limited defectiveness and complementation. J Virol 66:6587–6600. 10.1128/JVI.66.11.6587-6600.1992. 1404605 PMC240154

[59] Liao HX, Lynch R, Zhou T, Gao F, Alam SM, Boyd SD, Fire AZ, Roskin KM, Schramm CA, Zhang Z, Zhu J, Shapiro L, Program NCS, Mullikin JC, Gnanakaran S, Hraber P, Wiehe K, Kelsoe G, Yang G, Xia SM, Montefiori DC, Parks R, Lloyd KE, Scearce RM, Soderberg KA, Cohen M, Kamanga G, Louder MK, Tran LM, Chen Y, Cai F, Chen S, Moquin S, Du X, Joyce MG, Srivatsan S, Zhang B, Zheng A, Shaw GM, Hahn BH, Kepler TB, Korber BT, Kwong PD, Mascola JR, Haynes BF, NISC Comparative Sequencing Program. 2013. Co-evolution of a broadly neutralizing HIV-1 antibody and founder virus. Nature 496:469–476. 10.1038/nature12053. 23552890 PMC3637846

[60] Bonsignori M, Kreider EF, Fera D, Meyerhoff RR, Bradley T, Wiehe K, Alam SM, Aussedat B, Walkowicz WE, Hwang KK, Saunders KO, Zhang R, Gladden MA, Monroe A, Kumar A, Xia SM, Cooper M, Louder MK, McKee K, Bailer RT, Pier BW, Jette CA, Kelsoe G, Williams WB, Morris L, Kappes J, Wagh K, Kamanga G, Cohen MS, Hraber PT, Montefiori DC, Trama A, Liao HX, Kepler TB, Moody MA, Gao F, Danishefsky SJ, Mascola JR, Shaw GM, Hahn BH, Harrison SC, Korber BT, Haynes BF. 2017. Staged induction of HIV-1 glycan-dependent broadly neutralizing antibodies. Sci Transl Med 9:eaai7514. 10.1126/scitranslmed.aai7514. 28298420 PMC5562350

[61] Sanders RW, Derking R, Cupo A, Julien JP, Yasmeen A, de Val N, Kim HJ, Blattner C, de la Pena AT, Korzun J, Golabek M, de Los Reyes K, Ketas TJ, van Gils MJ, King CR, Wilson IA, Ward AB, Klasse PJ, Moore JP. 2013. A next-generation cleaved, soluble HIV-1 Env trimer, BG505 SOSIP.664 gp140, expresses multiple epitopes for broadly neutralizing but not non-neutralizing antibodies. PLoS Pathog 9:e1003618. 10.1371/journal.ppat.1003618. 24068931 PMC3777863

[62] Moore PL, Sheward D, Nonyane M, Ranchobe N, Hermanus T, Gray ES, Abdool Karim SS, Williamson C, Morris L. 2013. Multiple pathways of escape from HIV broadly cross-neutralizing V2-dependent antibodies. J Virol 87:4882–4894. 10.1128/JVI.03424-12. 23408621 PMC3624332

[63] Baalwa J, Wang S, Parrish NF, Decker JM, Keele BF, Learn GH, Yue L, Ruzagira E, Ssemwanga D, Kamali A, Amornkul PN, Price MA, Kappes JC, Karita E, Kaleebu P, Sanders E, Gilmour J, Allen S, Hunter E, Montefiori DC, Haynes BF, Cormier E, Hahn BH, Shaw GM. 2013. Molecular identification, cloning and characterization of transmitted/founder HIV-1 subtype A, D and A/D infectious molecular clones. Virology 436:33–48. 10.1016/j.virol.2012.10.009. 23123038 PMC3545109

[64] Li M, Salazar-Gonzalez JF, Derdeyn CA, Morris L, Williamson C, Robinson JE, Decker JM, Li Y, Salazar MG, Polonis VR, Mlisana K, Karim SA, Hong K, Greene KM, Bilska M, Zhou J, Allen S, Chomba E, Mulenga J, Vwalika C, Gao F, Zhang M, Korber BT, Hunter E, Hahn BH, Montefiori DC. 2006. Genetic and neutralization properties of subtype C human immunodeficiency virus type 1 molecular env clones from acute and early heterosexually acquired infections in Southern Africa. J Virol 80:11776–11790. 10.1128/JVI.01730-06. 16971434 PMC1642599

[65] Del Prete GQ, Ailers B, Moldt B, Keele BF, Estes JD, Rodriguez A, Sampias M, Oswald K, Fast R, Trubey CM, Chertova E, Smedley J, LaBranche CC, Montefiori DC, Burton DR, Shaw GM, Markowitz M, Piatak M, Jr., KewalRamani VN, Bieniasz PD, Lifson JD, Hatziioannou T. 2014. Selection of unadapted, pathogenic SHIVs encoding newly transmitted HIV-1 envelope proteins. Cell Host Microbe 16:412–418. 10.1016/j.chom.2014.08.003. 25211081 PMC4268878

[66] Boyd DF, Peterson D, Haggarty BS, Jordan AP, Hogan MJ, Goo L, Hoxie JA, Overbaugh J. 2015. Mutations in HIV-1 envelope that enhance entry with the macaque CD4 receptor alter antibody recognition by disrupting quaternary interactions within the trimer. J Virol 89:894–907. 10.1128/JVI.02680-14. 25378497 PMC4300673

[67] Phillips AN. 1996. Reduction of HIV concentration during acute infection: independence from a specific immune response. Science 271:497–499. 10.1126/science.271.5248.497. 8560262

[68] Nowak MA, Lloyd AL, Vasquez GM, Wiltrout TA, Wahl LM, Bischofberger N, Williams J, Kinter A, Fauci AS, Hirsch VM, Lifson JD. 1997. Viral dynamics of primary viremia and antiretroviral therapy in simian immunodeficiency virus infection. J Virol 71:7518–7525. 10.1128/JVI.71.10.7518-7525.1997. 9311831 PMC192098

[69] Ndhlovu ZM, Kamya P, Mewalal N, Kloverpris HN, Nkosi T, Pretorius K, Laher F, Ogunshola F, Chopera D, Shekhar K, Ghebremichael M, Ismail N, Moodley A, Malik A, Leslie A, Goulder PJ, Buus S, Chakraborty A, Dong K, Ndung'u T, Walker BD. 2015. Magnitude and kinetics of CD8+ T cell activation during hyperacute HIV infection impact viral set point. Immunity 43:591–604. 10.1016/j.immuni.2015.08.012. 26362266 PMC4575777

[70] Robb ML, Eller LA, Kibuuka H, Rono K, Maganga L, Nitayaphan S, Kroon E, Sawe FK, Sinei S, Sriplienchan S, Jagodzinski LL, Malia J, Manak M, de Souza MS, Tovanabutra S, Sanders-Buell E, Rolland M, Dorsey-Spitz J, Eller MA, Milazzo M, Li Q, Lewandowski A, Wu H, Swann E, O'Connell RJ, Peel S, Dawson P, Kim JH, Michael NL, RV 217 Study Team. 2016. Prospective study of Acute HIV-1 infection in adults in East Africa and Thailand. N Engl J Med 374:2120–2130. 10.1056/NEJMoa1508952. 27192360 PMC5111628

[71] Markowitz M, Louie M, Hurley A, Sun E, Di Mascio M, Perelson AS, Ho DD. 2003. A novel antiviral intervention results in more accurate assessment of human immunodeficiency virus type 1 replication dynamics and T-cell decay in vivo. J Virol 77:5037–5038. 10.1128/jvi.77.8.5037-5038.2003. 12663814 PMC152136

[72] Decker JM, Bibollet-Ruche F, Wei X, Wang S, Levy DN, Wang W, Delaporte E, Peeters M, Derdeyn CA, Allen S, Hunter E, Saag MS, Hoxie JA, Hahn BH, Kwong PD, Robinson JE, Shaw GM. 2005. Antigenic conservation and immunogenicity of the HIV coreceptor binding site. J Exp Med 201:1407–1419. 10.1084/jem.20042510. 15867093 PMC2213183

[73] Keele BF, Giorgi EE, Salazar-Gonzalez JF, Decker JM, Pham KT, Salazar MG, Sun C, Grayson T, Wang S, Li H, Wei X, Jiang C, Kirchherr JL, Gao F, Anderson JA, Ping LH, Swanstrom R, Tomaras GD, Blattner WA, Goepfert PA, Kilby JM, Saag MS, Delwart EL, Busch MP, Cohen MS, Montefiori DC, Haynes BF, Gaschen B, Athreya GS, Lee HY, Wood N, Seoighe C, Perelson AS, Bhattacharya T, Korber BT, Hahn BH, Shaw GM. 2008. Identification and characterization of transmitted and early founder virus envelopes in primary HIV-1 infection. Proc Natl Acad Sci U S A 105:7552–7557. 10.1073/pnas.0802203105. 18490657 PMC2387184

[74] Davis KL, Bibollet-Ruche F, Li H, Decker JM, Kutsch O, Morris L, Salomon A, Pinter A, Hoxie JA, Hahn BH, Kwong PD, Shaw GM. 2009. Human immunodeficiency virus type 2 (HIV-2)/HIV-1 envelope chimeras detect high titers of broadly reactive HIV-1 V3-specific antibodies in human plasma. JVI 83:1240–1259. 10.1128/JVI.01743-08. PMC2620909 19019969

[75] Del Prete GQ, Scarlotta M, Newman L, Reid C, Parodi LM, Roser JD, Oswald K, Marx PA, Miller CJ, Desrosiers RC, Barouch DH, Pal R, Piatak M, Jr., Chertova E, Giavedoni LD, O'Connor DH, Lifson JD, Keele BF. 2013. Comparative characterization of transfection- and infection-derived simian immunodeficiency virus challenge stocks for in vivo nonhuman primate studies. J Virol 87:4584–4595. 10.1128/JVI.03507-12. 23408608 PMC3624367

[76] Louder MK, Sambor A, Chertova E, Hunte T, Barrett S, Ojong F, Sanders-Buell E, Zolla-Pazner S, McCutchan FE, Roser JD, Gabuzda D, Lifson JD, Mascola JR. 2005. HIV-1 envelope pseudotyped viral vectors and infectious molecular clones expressing the same envelope glycoprotein have a similar neutralization phenotype, but culture in peripheral blood mononuclear cells is associated with decreased neutralization sensitivity. Virology 339:226–238. 10.1016/j.virol.2005.06.003. 16005039

[77] Cohen YZ, Lorenzi JCC, Seaman MS, Nogueira L, Schoofs T, Krassnig L, Butler A, Millard K, Fitzsimons T, Daniell X, Dizon JP, Shimeliovich I, Montefiori DC, Caskey M, Nussenzweig MC. 2017. Neutralizing activity of broadly neutralizing anti-HIV-1 antibodies against clade B clinical isolates produced in peripheral blood mononuclear cells. J Virol 92:e01883-17. 10.1128/JVI.01883-17. PMC5809738 29237833

[78] Patel P, Borkowf CB, Brooks JT, Lasry A, Lansky A, Mermin J. 2014. Estimating per-act HIV transmission risk: a systematic review. AIDS 28:1509–1519. 10.1097/QAD.0000000000000298. 24809629 PMC6195215

[79] Sok D, Burton DR. 2018. Recent progress in broadly neutralizing antibodies to HIV. Nat Immunol 19:1179–1188. 10.1038/s41590-018-0235-7. 30333615 PMC6440471

[80] Steichen JM, Lin YC, Havenar-Daughton C, Pecetta S, Ozorowski G, Willis JR, Toy L, Sok D, Liguori A, Kratochvil S, Torres JL, Kalyuzhniy O, Melzi E, Kulp DW, Raemisch S, Hu X, Bernard SM, Georgeson E, Phelps N, Adachi Y, Kubitz M, Landais E, Umotoy J, Robinson A, Briney B, Wilson IA, Burton DR, Ward AB, Crotty S, Batista FD, Schief WR. 2019. A generalized HIV vaccine design strategy for priming of broadly neutralizing antibody responses. Science 366:eaax4380. 10.1126/science.aax4380. 31672916 PMC7092357

[81] Kwong PD, Mascola JR. 2018. HIV-1 vaccines based on antibody identification, B cell ontogeny, and epitope structure. Immunity 48:855–871. 10.1016/j.immuni.2018.04.029. 29768174

[82] Haynes BF, Burton DR, Mascola JR. 2019. Multiple roles for HIV broadly neutralizing antibodies. Sci Transl Med 11:eaaz2686. 10.1126/scitranslmed.aaz2686. 31666399 PMC7171597

[83] Ou L, Kong WP, Chuang GY, Ghosh M, Gulla K, O'Dell S, Varriale J, Barefoot N, Changela A, Chao CW, Cheng C, Druz A, Kong R, McKee K, Rawi R, Sarfo EK, Schon A, Shaddeau A, Tsybovsky Y, Verardi R, Wang S, Wanninger TG, Xu K, Yang GJ, Zhang B, Zhang Y, Zhou T, Program VRCP, Arnold FJ, Doria-Rose NA, Lei QP, Ryan ET, Vann WF, Mascola JR, Kwong PD, VRC Production Program. 2020. Preclinical development of a fusion peptide conjugate as an HIV vaccine immunogen. Sci Rep 10:3032. 10.1038/s41598-020-59711-y. 32080235 PMC7033230

[84] Gibbs RA, Rogers J, Katze MG, Bumgarner R, Weinstock GM, Mardis ER, Remington KA, Strausberg RL, Venter JC, Wilson RK, Batzer MA, Bustamante CD, Eichler EE, Hahn MW, Hardison RC, Makova KD, Miller W, Milosavljevic A, Palermo RE, Siepel A, Sikela JM, Attaway T, Bell S, Bernard KE, Buhay CJ, Chandrabose MN, Dao M, Davis C, Delehaunty KD, Ding Y, Dinh HH, Dugan-Rocha S, Fulton LA, Gabisi RA, Garner TT, Godfrey J, Hawes AC, Hernandez J, Hines S, Holder M, Hume J, Jhangiani SN, Joshi V, Khan ZM, Kirkness EF, Cree A, Fowler RG, Lee S, Lewis LR, Li Z, Rhesus Macaque Genome Sequencing and Analysis Consortium, . 2007. Evolutionary and biomedical insights from the rhesus macaque genome. Science 316:222–234. 10.1126/science.1139247. 17431167

[85] Ramesh A, Darko S, Hua A, Overman G, Ransier A, Francica JR, Trama A, Tomaras GD, Haynes BF, Douek DC, Kepler TB. 2017. Structure and diversity of the rhesus macaque immunoglobulin loci through multiple de novo genome assemblies. Front Immunol 8:1407. 10.3389/fimmu.2017.01407. 29163486 PMC5663730

[86] Goonetilleke N, Liu MK, Salazar-Gonzalez JF, Ferrari G, Giorgi E, Ganusov VV, Keele BF, Learn GH, Turnbull EL, Salazar MG, Weinhold KJ, Moore S, BCC, Letvin N, Haynes BF, Cohen MS, Hraber P, Bhattacharya T, Borrow P, Perelson AS, Hahn BH, Shaw GM, Korber BT, McMichael AJ, CHAVI Clinical Core B. 2009. The first T cell response to transmitted/founder virus contributes to the control of acute viremia in HIV-1 infection. J Exp Med 206:1253–1272. 10.1084/jem.20090365. 19487423 PMC2715063

[87] Salazar-Gonzalez JF, Salazar MG, Keele BF, Learn GH, Giorgi EE, Li H, Decker JM, Wang S, Baalwa J, Kraus MH, Parrish NF, Shaw KS, Guffey MB, Bar KJ, Davis KL, Ochsenbauer-Jambor C, Kappes JC, Saag MS, Cohen MS, Mulenga J, Derdeyn CA, Allen S, Hunter E, Markowitz M, Hraber P, Perelson AS, Bhattacharya T, Haynes BF, Korber BT, Hahn BH, Shaw GM. 2009. Genetic identity, biological phenotype, and evolutionary pathways of transmitted/founder viruses in acute and early HIV-1 infection. J Exp Med 206:1273–1289. 10.1084/jem.20090378. 19487424 PMC2715054

[88] Wyatt R, Kwong PD, Desjardins E, Sweet RW, Robinson J, Hendrickson WA, Sodroski JG. 1998. The antigenic structure of the HIV gp120 envelope glycoprotein. Nature 393:705–711. 10.1038/31514. 9641684

[89] Starcich BR, Hahn BH, Shaw GM, McNeely PD, Modrow S, Wolf H, Parks ES, Parks WP, Josephs SF, Gallo RC. 1986. Identification and characterization of conserved and variable regions in the envelope gene of HTLV-III/LAV, the retrovirus of AIDS. Cell 45:637–648. 10.1016/0092-8674(86)90778-6. 2423250

[90] Kwong PD, Doyle ML, Casper DJ, Cicala C, Leavitt SA, Majeed S, Steenbeke TD, Venturi M, Chaiken I, Fung M, Katinger H, Parren PW, Robinson J, Van Ryk D, Wang L, Burton DR, Freire E, Wyatt R, Sodroski J, Hendrickson WA, Arthos J. 2002. HIV-1 evades antibody-mediated neutralization through conformational masking of receptor-binding sites. Nature 420:678–682. 10.1038/nature01188. 12478295

[91] Wei X, Decker JM, Wang S, Hui H, Kappes JC, Wu X, Salazar-Gonzalez JF, Salazar MG, Kilby JM, Saag MS, Komarova NL, Nowak MA, Hahn BH, Kwong PD, Shaw GM. 2003. Antibody neutralization and escape by HIV-1. Nature 422:307–312. 10.1038/nature01470. 12646921

[92] Holmes EC. 2003. Error thresholds and the constraints to RNA virus evolution. Trends Microbiol 11:543–546. 10.1016/j.tim.2003.10.006. 14659685 PMC7172642

[93] Woo J, Robertson DL, Lovell SC. 2010. Constraints on HIV-1 diversity from protein structure. J Virol 84:12995–13003. 10.1128/JVI.00702-10. 20881050 PMC3004343

[94] Haddox HK, Dingens AS, Bloom JD. 2016. Experimental estimation of the effects of all amino-acid Mutations to HIV's envelope protein on viral replication in cell culture. PLoS Pathog 12:e1006114. 10.1371/journal.ppat.1006114. 27959955 PMC5189966

[95] Moore PL. 2018. The neutralizing antibody response to the HIV-1 Env protein. Curr HIV Res 16:21–28. 10.2174/1570162X15666171124122044. 29173180 PMC6234226

[96] Han Q, Jones JA, Nicely NI, Reed RK, Shen X, Mansouri K, Louder M, Trama AM, Alam SM, Edwards RJ, Bonsignori M, Tomaras GD, Korber B, Montefiori DC, Mascola JR, Seaman MS, Haynes BF, Saunders KO. 2019. Difficult-to-neutralize global HIV-1 isolates are neutralized by antibodies targeting open envelope conformations. Nat Commun 10:2898. 10.1038/s41467-019-10899-2. 31263112 PMC6602974

[97] Tartaglia LJ, Gupte S, Pastores KC, Trott S, Abbink P, Mercado NB, Li Z, Liu PT, Borducchi EN, Chandrashekar A, Bondzie EA, Hamza V, Kordana N, Mahrokhian S, Lavine CL, Seaman MS, Li H, Shaw GM, Barouch DH. 2020. Differential outcomes following optimization of simian-human immunodeficiency viruses from clades AE, B, and C. J Virol 94:e01860-19. 10.1128/JVI.01860-19. 32132241 PMC7199416

[98] Keele BF, Li H, Learn GH, Hraber P, Giorgi EE, Grayson T, Sun C, Chen Y, Yeh WW, Letvin NL, Mascola JR, Nabel GJ, Haynes BF, Bhattacharya T, Perelson AS, Korber BT, Hahn BH, Shaw GM. 2009. Low-dose rectal inoculation of rhesus macaques by SIVsmE660 or SIVmac251 recapitulates human mucosal infection by HIV-1. J Exp Med 206:1117–1134. 10.1084/jem.20082831. 19414559 PMC2715022

[99] Deleage C, Immonen TT, Fennessey CM, Reynaldi A, Reid C, Newman L, Lipkey L, Schlub TE, Camus C, O'Brien S, Smedley J, Conway JM, Del Prete GQ, Davenport MP, Lifson JD, Estes JD, Keele BF. 2019. Defining early SIV replication and dissemination dynamics following vaginal transmission. Sci Adv 5:eaav7116. 10.1126/sciadv.aav7116. 31149634 PMC6541462

[100] Bar KJ, Coronado E, Hensley-McBain T, O’Connor MA, Osborn JM, Miller C, Gott TM, Wangari S, Iwayama N, Ahrens CY, Smedley J, Moats C, Lynch RM, Haddad EK, Haigwood NL, Fuller DH, Shaw GM, Klatt NR, Manuzak JA. 2019. Simian-human immunodeficiency virus SHIV.CH505 infection of rhesus macaques results in persistent viral replication and induces intestinal immunopathology. J Virol 93:e00372-19. 10.1128/JVI.00372-19. 31217249 PMC6714786

[101] Bauer AM, Ziani W, Lindemuth E, Kuri-Cervantes L, Li H, Lee FH, Watkins M, Ding W, Xu H, Veazey R, Bar KJ. 2020. Novel transmitted/founder simian-human immunodeficiency viruses for human immunodeficiency virus latency and cure research. J Virol 94:e01659. 10.1128/JVI.01659-19. 31969435 PMC7108852

[102] Sarzotti-Kelsoe M, Bailer RT, Turk E, Lin CL, Bilska M, Greene KM, Gao H, Todd CA, Ozaki DA, Seaman MS, Mascola JR, Montefiori DC. 2014. Optimization and validation of the TZM-bl assay for standardized assessments of neutralizing antibodies against HIV-1. J Immunol Methods 409:131–146. 10.1016/j.jim.2013.11.022. 24291345 PMC4040342

[103] Song H, Giorgi EE, Ganusov VV, Cai F, Athreya G, Yoon H, Carja O, Hora B, Hraber P, Romero-Severson E, Jiang C, Li X, Wang S, Li H, Salazar-Gonzalez JF, Salazar MG, Goonetilleke N, Keele BF, Montefiori DC, Cohen MS, Shaw GM, Hahn BH, McMichael AJ, Haynes BF, Korber B, Bhattacharya T, Gao F. 2018. Tracking HIV-1 recombination to resolve its contribution to HIV-1 evolution in natural infection. Nat Commun 9:1928. 10.1038/s41467-018-04217-5. 29765018 PMC5954121

